# Small Theropod Teeth from the Late Cretaceous of the San Juan Basin, Northwestern New Mexico and Their Implications for Understanding Latest Cretaceous Dinosaur Evolution

**DOI:** 10.1371/journal.pone.0093190

**Published:** 2014-04-07

**Authors:** Thomas E. Williamson, Stephen L. Brusatte

**Affiliations:** 1 New Mexico Museum of Natural History and Science, Albuquerque, New Mexico, United States of America; 2 School of GeoSciences, University of Edinburgh, Edinburgh, Scotland, United Kingdom; University of Pennsylvania, United States of America

## Abstract

Studying the evolution and biogeographic distribution of dinosaurs during the latest Cretaceous is critical for better understanding the end-Cretaceous extinction event that killed off all non-avian dinosaurs. Western North America contains among the best records of Late Cretaceous terrestrial vertebrates in the world, but is biased against small-bodied dinosaurs. Isolated teeth are the primary evidence for understanding the diversity and evolution of small-bodied theropod dinosaurs during the Late Cretaceous, but few such specimens have been well documented from outside of the northern Rockies, making it difficult to assess Late Cretaceous dinosaur diversity and biogeographic patterns. We describe small theropod teeth from the San Juan Basin of northwestern New Mexico. These specimens were collected from strata spanning Santonian – Maastrichtian. We grouped isolated theropod teeth into several morphotypes, which we assigned to higher-level theropod clades based on possession of phylogenetic synapomorphies. We then used principal components analysis and discriminant function analyses to gauge whether the San Juan Basin teeth overlap with, or are quantitatively distinct from, similar tooth morphotypes from other geographic areas. The San Juan Basin contains a diverse record of small theropods. Late Campanian assemblages differ from approximately co-eval assemblages of the northern Rockies in being less diverse with only rare representatives of troodontids and a *Dromaeosaurus*-like taxon. We also provide evidence that erect and recurved morphs of a *Richardoestesia*-like taxon represent a single heterodont species. A late Maastrichtian assemblage is dominated by a distinct troodontid. The differences between northern and southern faunas based on isolated theropod teeth provide evidence for provinciality in the late Campanian and the late Maastrichtian of North America. However, there is no indication that major components of small-bodied theropod diversity were lost during the Maastrichtian in New Mexico. The same pattern seen in northern faunas, which may provide evidence for an abrupt dinosaur extinction.

## Introduction

The Late Cretaceous terrestrial vertebrate record of western North America is among the best in the world and gives critical information on dinosaur taxonomic and morphological diversity over the final ∼20 million years of the Mesozoic. The Late Cretaceous is also the time when dinosaurs approached the end-Cretaceous extinction event, and dinosaur faunal dynamics leading up to this event may reveal important clues about the role the Chicxulub impact and other factors played in the ultimate extinction of non-avian dinosaurs [1], [2], [3], [4], [5]. Also, because this record spans a large geographic area of the Western Interior and a wide range of latitude (∼20 degrees), it has also been an important source for hypotheses regarding Late Cretaceous dinosaur biogeography [6], [7], [8], [9], [10] and beta diversity [11].

Estimates of dinosaur diversity are hindered in part because of sampling biases in the fossil record. Such biases are unfortunately pervasive in the latest Cretaceous (Campanian-Maastrichtian) record. One of the great difficulties of studying how dinosaurs changed during the ∼20 million years before their extinction is that only North America preserves a well-sampled, well-dated succession of stratigraphically stacked dinosaur faunas from this time [12], [13], [14]. With that said, however, much of our understanding of dinosaur diversity from the latest Cretaceous of North America actually comes from study of a relatively small geographic area, as it is based primarily on the extremely rich fossil faunas of the northern Rocky Mountain region, including faunas of the Campanian Belly River Group and Horseshoe Canyon Formation of southern Alberta, the Judith River group of Montana, and the Lance and Hell Creek formations of Montana and nearby states. Dinosaur-bearing units from outside of this region tend to be much less fossiliferous, less studied, and more poorly known, hampering understanding of both dinosaur biogeography and diversity.

Additional sampling biases affect estimates of the diversity of small dinosaurs from the Late Cretaceous of western North America, even from the best studied and sampled sites. Small-bodied dinosaurs are particularly underrepresented in the fossil record and remain poorly known even after more than a century of exploration (see also [15], [16]). Most of the fossil evidence for small theropod dinosaurs in fossil faunas of the Western Interior are from isolated teeth [10], [17], [18], [19], [20], [21], [22], [23] which were typically recovered from microvertebrate fossil concentrations using screenwashing techniques. Amazingly, it was only very recently that the first diagnostic skeletal remains of a North American Maastrichtian dromaeosaurid were recovered, despite the recovery of thousands of teeth of these animals over the past several decades [24].

The rarity of skeletal remains of small theropods, even in these well-sampled faunas, speaks to the importance of isolated teeth as the primary evidence for understanding the diversity and evolution of carnivorous dinosaurs during the run-up to their extinction. Frustratingly, some recent studies suggest that small isolated theropod teeth are probably only diagnostic at a low taxonomic level [10], [20]. Nonetheless, because they typically are the only representation of small theropods in many faunas, teeth have continued to be used as proxies for small theropod diversity in those faunas. More promising, recent studies of specimen-rich theropod tooth datasets using various multivariate statistical methods suggest that some small theropod teeth can be quantitatively distinguished from each other, offering great potential for future studies of small theropod diversity over time and space based on isolated teeth (e.g., [17]).

Here we document isolated small theropod teeth from the San Juan Basin of northwestern New Mexico. The San Juan Basin contains some of the richest Late Cretaceous terrestrial faunas from outside of the northern Rocky Mountain region and is thus important for understanding Late Cretaceous biogeography and faunal heterogeneity across western North America during this time. While numerous Late Cretaceous assemblages of small theropod teeth have been described from the northern Rockies in recent years, few studies have assessed or included well-documented small theropod teeth from southern Late Cretaceous vertebrate faunas [18], [25]. Instead, most reports of small theropod taxa based on isolated teeth have appeared only in faunal lists, abstracts, as brief and undetailed descriptions, or in unpublished theses [26], [27], [28], [29], [30], [31], [32], [33]. This makes it unclear whether southern North American theropod faunas were different from those in the north during the latest Cretaceous, and hinders our ability to use the southern record to better understand dinosaur diversity changes before the end-Cretaceous extinction.

The San Juan Basin terrestrial vertebrate record spans from Santonian – Maastrichtian time and contains among the very few records of dinosaurs from the Santonian and early Campanian of western North America [12], [34], [35], [36], making it an important (and in some cases unique) record of dinosaur diversity during the middle part of the Late Cretaceous. In addition, recent studies indicate that the Alamo Wash local fauna of the San Juan Basin is of latest Cretaceous age [37], which would make it one the most diverse known latest Cretaceous terrestrial vertebrate faunas from outside of the northern Rocky Mountain region [12], and therefore a critical fauna for understanding the dinosaur extinction. Teams from the New Mexico Museum of Natural History and Science, led by TEW, have been collecting theropod teeth and other specimens from the San Juan Basin for many years employing underwater screenwashing techniques, and for the first time we fully document these collections here and discuss their implications for understanding dinosaur diversity, evolution, biogeography, and extinction.

## Materials and Methods

With the exception of one specimen housed at the University of Kansas Museum of Paleontology (KUVP), all specimens described here are accessioned into the Geoscience Collections of the New Mexico Museum of Natural History and Science (NMMNH), an institution accredited by the American Association of Museums. Access to precise locality information is restricted to qualified researchers and land management personnel.

All specimens described in this study were collected under permits obtained from the United States Department of the Interior's Bureau of Land Management (BLM).

### Tooth identification

Many previous studies have referred isolated theropod teeth to a particular genus and species. Some of these studies were based on specimens from the Dinosaur Park Formation of southern Alberta, where isolated teeth could be compared with those associated with diagnostic partial skulls and skeletons collected from the same stratum (e.g., [21], [38]). Following this, many workers have referred isolated teeth from widely geographically separated faunas and/or faunas of different ages to many of the same genera and species [18], [22], [39]. In recent years, several workers have suggested that many, if not most, isolated theropod teeth may not be diagnostic to species or genus level (e.g., [17], [40], [41]). Also, several workers have concluded that it is unlikely that single taxa would be present over the large geographic areas represented by western North America and the long intervals spanning many millions of years (e.g., [17], [23], [42]). In addition, it is unclear how much individual variation of tooth morphology occurs within some taxa. Therefore, caution should be exercised in referring isolated teeth to any particular theropod taxon.

Here, we do not not assign teeth to specific genera or species of theropods known from skeletal material, but rather group the specimens into informal morphotypes based on the shared possession of features which have previously been used to diagnose morphotypes in the literature (e.g., [10], [17], [20], [23]).

These morphotypes were assigned to specific phylogenetic groupings of theropods based on three lines of evidence. First, we searched for explicit discrete characters that have been found to unite clades in phylogenetic analyses. To do so we used the tyrannosauroid-specific analysis of Brusatte et al. [43] to assess tyrannosauroid (and ingroup) affinities and the derived coelurosaurian analysis of Turner et al. [16], which was modified to include a larger sample of coelurosaurs by Brusatte [44], to assess affinities of various coelurosaurian subgroups. The possession of phylogenetic synapomorphies is a very strong line of evidence that a certain tooth, or collection of teeth, can be assigned to a certain theropod group.

Second, we performed a principal components analysis (PCA), a multivariate technique that takes a number of measurements for a sample of teeth and distills them into a smaller and more manageable set of axes describing the primary variability among the specimens. This allows the teeth to be plotted in a morphospace, which can be visually inspected and assessed statistically to see if the San Juan Basin teeth overlap with teeth referred to certain groups (e.g., Dromaeosauridae, Troodontidae) from other geographic areas. We added the San Juan Basin teeth to the recently published dataset of Larson and Currie [17], which included measurement data for over 1200 small theropod teeth mainly from the latest Cretaceous of the northern Rockies region. Each tooth in the dataset is scored for five standard measurements: Fore-aft basal length (FABL), crown height (CH), basal width (BW), mesial denticles per mm (ADM), and distal denticles per mm (PDM). Those San Juan Basin teeth that could be assessed for only one or two of these measurements were excluded from the analysis, as PCA is sensitive to missing data. The analysis was performed in PAST [45] with missing data cells estimated by average column substitution.

PCA was not used to test the affinities of San Juan Basin teeth identified as tyrannosauroids (based on discrete phylogenetic characters) because of concerns about ontogenetic variation. It is widely known that large-bodied tyrannosauroids underwent extreme morphological changes during ontogeny, including drastic changes in the proportions and thickness of their teeth. This poses a problem for PCAs because the analysis will simply group specimens based on measurements, meaning that juvenile and adult teeth are likely to cluster separately in morphospace. Differences between juvenile and adult specimens of the same taxon may often be greater than differences between adults of separate taxa, making it extremely difficult to tease apart ontogenetic and taxonomic variation in a PCA without an independent age indicator of the teeth in question (e.g., [40], [46]). This is possible with in situ dentitions, which can be aged based on histological growth line data from other parts of the skeleton, but not with isolated teeth. This ontogenetic issue has been shown to affect previous PCAs of tyrannosauroid teeth (e.g., [40], [46], [47]).

Third, we performed a series of discriminant function analyses (DFA) as a heuristic tool for assessing whether certain San Juan Basin tooth morphotypes are quantitatively distinct from similar morphotypes from more northern regions, whose taxonomic identities are better constrained (and in some cases clearly constrained by synapomorphies). DFA works by first dividing a sample into two groups (in this case, San Juan Basin teeth vs. teeth from another region), calculating a multivariate mean (group centroid) for the two groups, and then reclassifying the individual teeth based on their distances to the centroids (e.g., assessing whether each tooth is closer to the centroid of group 1 or group 2). The original classification is compared to the new classification and a hit ratio is calculated: the percentage of specimens that are correctly assigned to their group by the DFA [17], [48]. Hammer and Harper [48] consider a hit ratio of above 90% to be sufficient for demonstrating that the two groups are distinct. Following this line of reasoning, if we observe a hit ratio less than 90% this means that the two groups are not clearly quantitatively distinct, which provides evidence in this case that the San Juan Basin teeth can be assigned to the same type of group as the more firmly identified teeth from elsewhere. We reiterate that this is not a conclusive statistical test, but an exploratory tool that we use in conjunction with the much more rigorously grounded synapomorphy-based approach and PCA to explore structure in our data and assess whether the San Juan Basin teeth are generally similar (or not) to teeth from elsewhere.

We peformed a series of DFAs in PAST, comparing several San Juan Basin morphotypes to a morphotype from more northern regions that is assumed to be roughly equivalent, based on shared possession of characters (in some cases synapomorphies) and the literature (e.g., [10], [17], [20], [21], [23], [38]). Data for the northern teeth were taken from Larson and Currie [17]. Each DFA was run twice: first with all San Juan Basin teeth included and second by excluding those teeth that could be scored for only one or two of the five total measurements.

### Assessing differences between San Juan Basin teeth and other samples

Our primary focus in this study is to identify distinct morphotypes of small theropod teeth from the San Juan Basin and assign these morphotypes to higher-level groups of theropods (see above). In some cases, however, we are also interested in testing whether the morphotypes from the San Juan Basin are quantitatively distinct from similar morphotypes (assumed to be similar taxa) in more northern regions.

We used two methods to test for differences. First, we used DFAs (see above), with the rationale that a hit ratio of more than 90% is good evidence that the two groups are quantitatively distinct [48]. Second, in specific cases we tested for distinct differences between groups in morphospace, based on the PCA. We performed a two-group permutation test in PAST to assess significant statistical differences between two *a priori* defined groups in the morphospace based on all axes. The test assesses equality of the means of the two groups, by comparing the observed difference between the means of the two samples in morphospace with a distribution of group mean differences constructed from 2000 random permutations. Note that a significant result indicating separation of two groups does not necessarily mean that the two groups do not belong to the same clade, or even the same species or morphotype, as Larson and Currie [17] have shown the teeth of the same morphotype are often significantly different depending on age and geography.

### Geologic Setting

**Figure 1:**
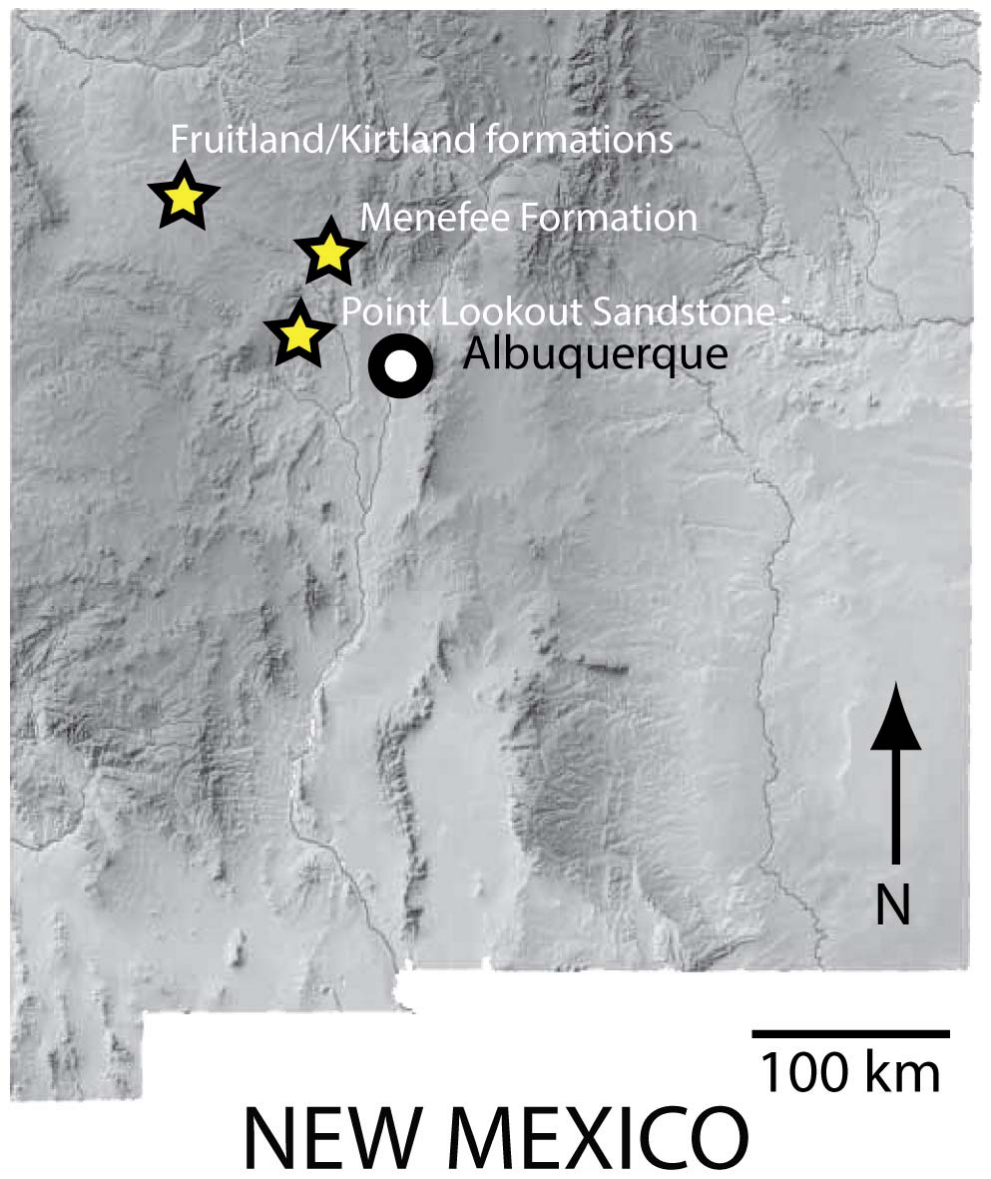
Map of New Mexico showing the location of the locales where small theropod teeth were collected.

**Figure 2:**
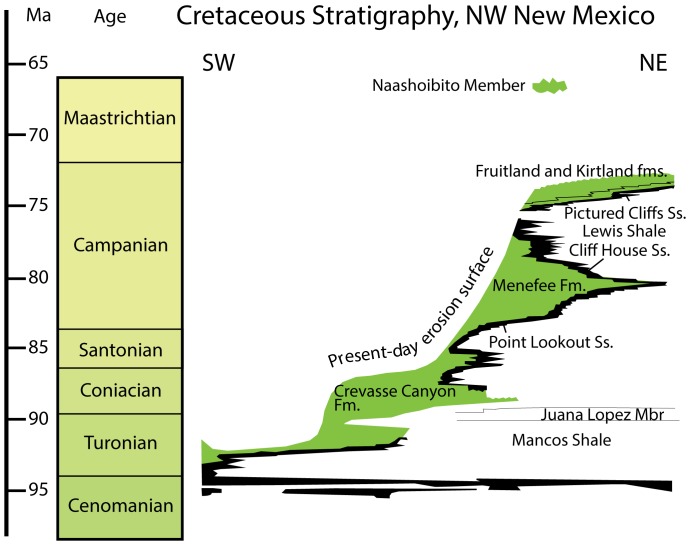
Stratigraphic distribution of Late Cretaceous small theropod teeth of New Mexico. 1, Hosta Tongue of the Point Lookout Sandstone (L-297); 2, Allison Member, Menefee Formation (L-3034); 3, Fossil Forest Member, Fruitland Formation (L-1882, 3117, 4062, 4063, 4256, 4276, 4718, 6266) and Hunter Wash Member (L-1708, 3490), Farmington Sandstone Member (), and De-na-zin Member (L-1610, 3228, 3532, 4722), Kirtland Formation; 4, Naashoibito Member (L-4005). Time scale is after Gradstein et al. [56].

**Figure 3:**
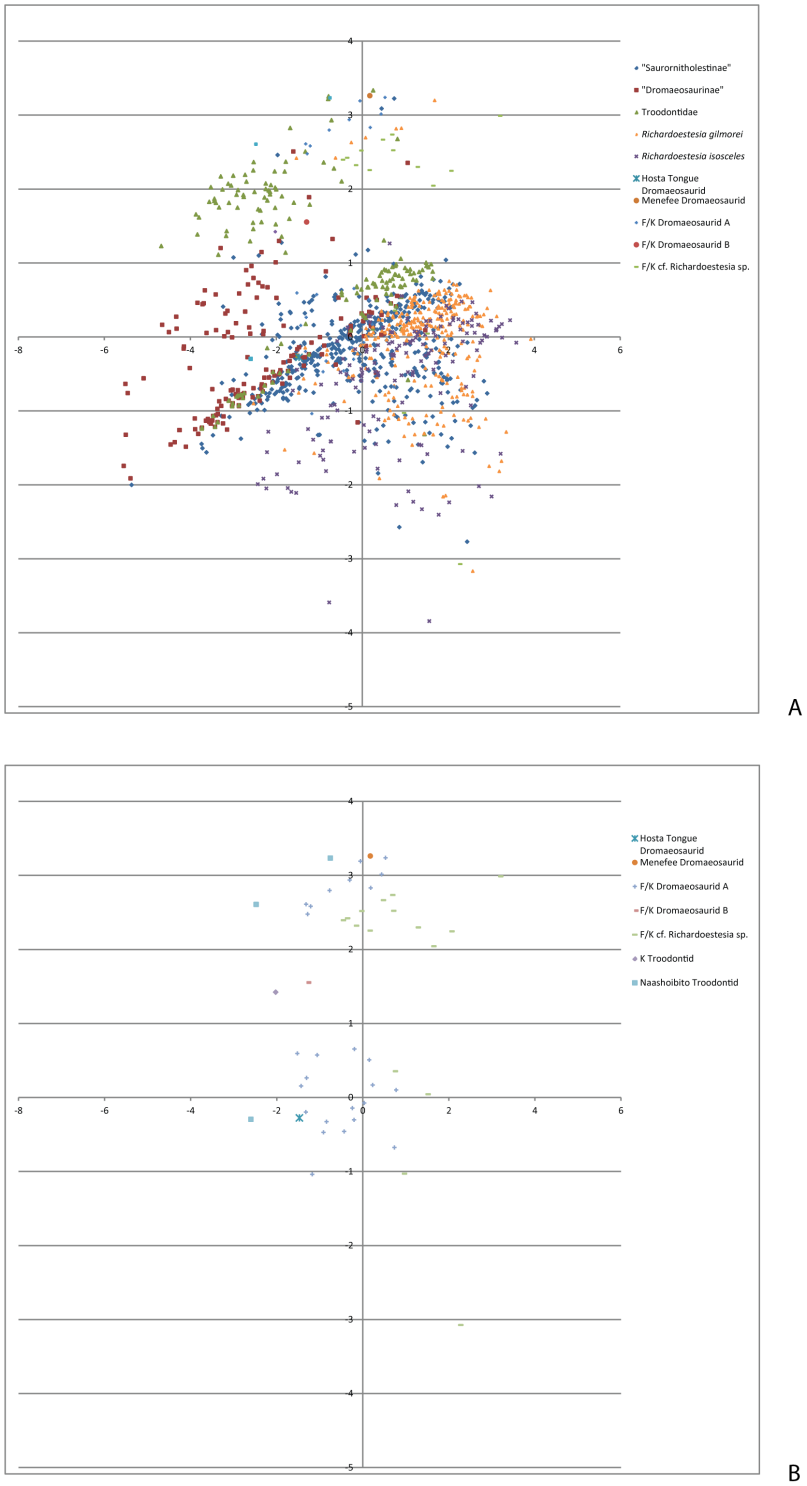
Principal components analysis of Late Cretaceous small theropod teeth based on data in the Supplementary Information (Appendices S1, S2). A, Full PCA of a dataset including small theropod teeth compiled by Larson and Currie [17] from several Late Cretaceous locales of western North America and specimens from the San Juan Basin, New Mexico; B, Simplified version of the PCA plot depicting only small theropod teeth from the San Juan Basin, New Mexico, for clarity (this is not based on a separate analysis, but is the same as Plot A but with the non-San Juan Basin specimens not shown). Summary statistics (e.g., eigenvalues and PC coefficients) are given in Appendix S2.

**Figure 4:**
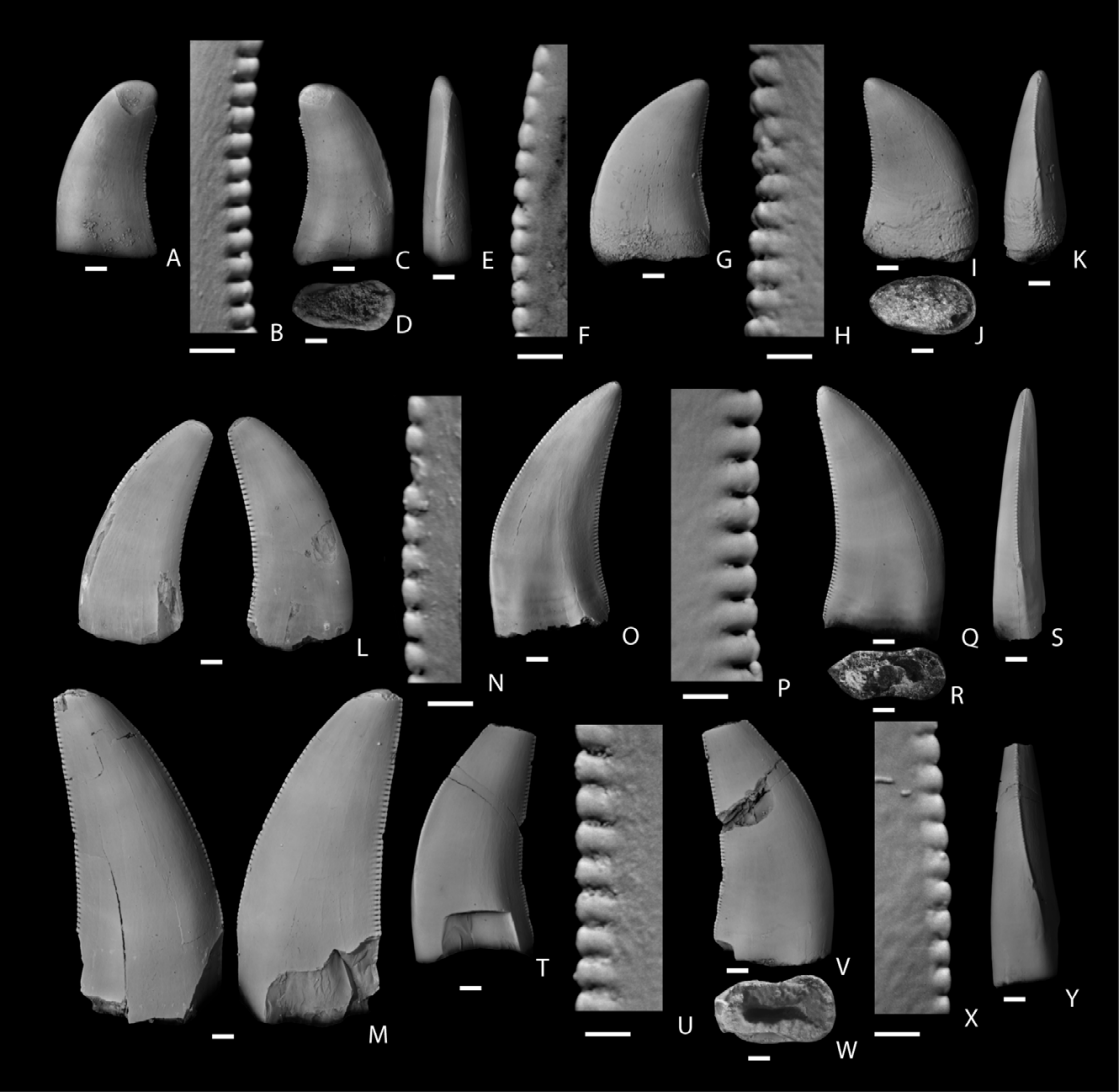
Small Tyrannosauroidea teeth from the San Juan Basin, New Mexico. A–K, teeth of cf. Tyrannosauroidea from the Santonian Hosta Tongue, Point Lookout Sandstone. A–E, NMMNH P-27484, in labial (A), labial side of distal carina (B), lingual (C), basal (D), and mesial (E) views; F–K, NMMNH P-27485, lingual side of mesial carina (F), lingual (G), labial side of distal carina (H), labial (I), basal (J), and mesial (K) views. L–S, small tyrannosauroid teeth from the upper Campanian De-na-zin Member, Kirtland Formation. L–M, NMMNH P-33903 two associated shed teeth in lingual (L) and labial (M) views; N–S, NMMNH P-27280, lingual side of mesial carina (N), lingual (O), lingual side of distal carina (P), labial (Q), basal (R), and mesial (S) views; T–Y, small tyrannosauroid teeth from the upper Maastrichtian Naashoibito Member, Kirtland Formation, NMMNH P-32567, lingual (T), labial side of distal carina (U), labial (V), basal (W), labial side of mesial carina (X), and mesial (Y) views. The scale bar below each image is 1 mm long.

Most of the teeth reported here were retrieved from sediments deposited in coastal and alluvial plain settings near the western margin of the Western Interior Seaway in what is now northwestern New Mexico (Figs. 1–2). The oldest samples reported here are from the Santonian Hosta Tongue of the Point Lookout Sandstone (Fig. 1; 1), a single tooth is from the lower Campanian Menefee Formation (Fig. 1; 2), the largest samples of teeth are from the upper Campanian Fruitland and lower Kirtland formations (Fig. 1; 3), and a small but significant sample is from the upper Maastrichtian Naashoibito Member, Kirtland Formation (Fig. 1; 4).

#### Hosta Tongue, Point Lookout Sandstone

Four small theropod teeth are reported from NMMNH locality 297, which is located along the western side of the Rio Puerco Valley about 35 km west of Albuquerque. Bourdon et al. [49] reported that L-297 is ∼6.5 m below the local top of the ∼45 m thick Hosta Tongue. Molenaar [50] concluded that the Hosta Tongue in the southern San Juan Basin is middle Santonian in age (see [49]). The paleoenvironmental has been described as fluvio-deltaic to offshore sandbar, or beach [49]. Fossils from locality L-297 are a mix of marine and nonmarine taxa that includes chondrichthyans, turtles, mosasaurs, plesiosaurs, and dinosaurs [51], [52], [53], [54].

#### Allison Member, Menefee Formation

A single small theropod tooth (NMMNH P-25054) from the Allison Member of the Menefee Formation was found during preparation of a centrosaurine ceratopsian and was referred to cf. *Saurornitholestes* sp. by Williamson [35]. The Menefee Formation was deposited as part of a clastic wedge that prograded northeastward in Santonian through early Campanian time ([50], Fig. 2). Radiometric dating of volcanic ashes from near the top of the Menefee Formation in the eastern part of the San Juan Basin place an upper limit on the vertebrate fauna of 78±0.26 Ma [55], which is middle Campanian in age [56]. The Allison Member represents a coastal plain environment with high sinuousity streams. A meager vertebrate fauna described from the Allison Formation of the eastern San Juan Basin includes the alligatoroid *Brachychampsa sealeyi* [57] and an unidentified centrosaurine ceratopsian [35]. In addition there are preliminary reports of richer vertebrate faunas [34], [58].

#### Fruitland and lower Kirtland formations

These sediments were deposited landward of the regressing western shoreline of the Western Interior Seaway toward the end of the Campanian. They represent an increasingly more landward succession of depositional environments, from a deltaic complex landward of the shoreline of the Western Interior Seaway (Fossil Forest Member, Fruitland Formation) to an alluvial floodplain with high sinuosity streams (Hunter Wash and Farmington members, Kirtland Formation), and finally a well-drained alluvial floodplain with low sinuousity streams (De-na-zin Member, Kirtland Formation). The vertebrate faunas of the upper Fruitland (Fossil Forest Member) and lower Kirtland (Hunter Wash Member, Farmington, and De-na-zin members) are the most diverse non-marine vertebrate faunas of the Late Cretaceous of New Mexico. The vertebrate fauna from the upper Fruitland and Hunter Wash members of the mid-central San Juan Basin are collectively referred to as the Hunter Wash local fauna [59]. The vertebrate fauna of the De-na-zin Member, Kirtland Formation is referred to as the Willow Wash local fauna [60].

Microvertebrate sites are relatively abundant in the Fossil Forest Member, Fruitland Formation [30], [31], [61], [62], [63], [64], [65], [66], but few have been described from the lower Kirtland Formation [66].

#### Age of the Fruitland and Kirtland Formations (excluding the Naashoibito Member)

Radiometric dating (^40^Ar/^39^Ar) of sanidines from several altered volcanic ash beds through the Fruitland and Kirtland formations [67], [68] provide absolute dates that constrain the vertebrate faunas of the upper Fruitland and lower Kirtland formations. The radiometric dates range from 75.56±0.41 Ma. to 73.04±0.25 Ma (Fassett, 2009) placing these faunas in the late Campanian [56]. Fassett and Steiner's [67] Ash 4, which is near the Fruitland and Kirtland contact in the Hunter Wash area, is dated at 74.55±0.62 Ma (recalibrated age from Fassett and Steiner, 1997 as published in Fassett, 2009 with original error bars). This is stratigraphically within beds that produce the Hunter Wash local fauna. Therefore, the Hunter Wash local fauna is younger than the main fossiliferous intervals of the Judith River and Two Medicine formations of Montana and the Kaiparowits Formation of Utah (see [6], fig. 2; [13]). It is also approximately the same age as the Aguja Formation, which is thought to closely straddle the geomagnetic polarity chron C32/C33 boundary [69], which is about 74.3 Ma [70]. The top of the De-na-zin Member and the minimum age for the Willow Wash local fauna is constrained by Fassett and Steiner's [67] Ash J which is located near the top of the De-na-zin Member in the Hunter Wash area and dated at 73.04±0.25 Ma [68]. It is nearly one million years younger than the top of the Judith River Group of Alberta, the Two Medicine Formation, and the Kaiparowits Formation.

#### Biochronologic age of the Hunter Wash local fauna

Lucas and Sullivan [71], [72] introduced the “Kirtlandian land-vertebrate ‘age’”, a biochronological unit based on the vertebrate fossil assemblages from the upper Fruitland and Kirtland formations of the San Juan Basin. Sullivan and Lucas [71], [72] argued that this filled a temporal gap present between Russell's [73], [74] Cretaceous mammalian assemblages, the Judithian and the “Edmontonian” (used in parentheses here following Cifelli et al. [36]; see [36] for the most recent review). Russell [73], [74] had originally proposed the “Edmontonian” to fill a large gap between typical Judithian and Lancian age faunas (see [75]). However, as Woodburne [76] argued, the “Kirtlandian land-vertebrate ‘age’” is not explicitly defined on mammals, as are the North American Land Mammal Ages (NALMA) (see [77]) and it is best to keep such chronological systems independent as otherwise they may mask independent evolutionary patterns [76]. The “Edmontonian” age remains a poorly understood interval and a paleontological criterion for recognition of a Judithian/“Edmontonian” boundary has not yet been established [36], [78]. Cifelli et al. [36], Kielan-Jaworowska and others [79], and Wilson [80] concluded that the Hunter Wash local fauna is Judithian in age.

#### Naashoibito Member, Kirtland Formation

The Naashoibito Member [81] is a rock unit that is up to about 25 m thick and exposed over a relatively small geographic area between the head of Hunter Wash and Betonnie-Tsosie Wash along the southwestern edge of the San Juan Basin, a total distance of about 30 km [82], [83]. The Naashoibito typically includes a basal conglomerate, but this has been removed by scour at the base of overlying sandstones in some places. Above the basal conglomerate is a series of and purple and gray mudstones and clay-rich white sandstones, often with brown cannon-ball concretions [82]. The unit varies considerably in thickness due to erosional scour at the base of the overlying Ojo Alamo Sandstone (used here in a restricted sense following [81]). Lehman [82] interpreted the basal conglomerate to represent a thin sheet of coarse braided-stream alluvium which was first incised and then filled by sinuous channels and overbank deposits of an aggrading floodplain. The brightly banded mudstones represent mature paleosols that may have undergone intermittent, possibly seasonal, drying [82].

The Naashoibito Member contains the Alamo Wash Local fauna [84], a vertebrate fauna that includes a mostly fragmentary, yet relatively diverse, assemblage of taxa (see [85]) for a recent review). The Naashoibito Member has yielded microvertebrates including the teeth of small theropod dinosaurs [30], [31], [66], [84], [86], [87]. However, most reports of these faunas were preliminary or describe only mammalian specimens.

The Naashoibito is considered a member of the Ojo Alamo Sandstone by some authors (e.g., [68], [71], [72], [88]) and a member of the Kirtland Formation by others (e.g., [81], [87], [89], [90]). We follow Baltz et al. [81] in considering the Naashiobito to be part of the Kirtland Formation in part because it is lithologically distinct from the Kimbeto Member ( = Ojo Alamo Sandstone in the restricted sense of Baltz et al. [81]) and more closely resembles the underlying Kirtland Formation [82].

The upper limit to the age of the Naashoibito Member is constrained by the age of the overlying base of the Ojo Alamo Formation (sensu stricto) and the Nacimiento Formation. The age of the Ojo Alamo Formation is Paleocene based on pollen collected from it in the southeastern part of the San Juan Basin [91]. In the area of Barrel Springs, the base of the Nacimiento Formation preserves a narrow zone of reversed polarity that correlates with Chron 29r [37], [92]. Vertebrate fossils of early Paleocene age (middle and late Puercan North American Land Mammal ages) [93] occur near the base of the Nacimiento Formation within a normal polarity zone correlated with Chron 29n [92].

The age of the Naashoibito Member and the Alamo Wash local fauna is contentious, and recent age estimates range from late Campanian or early Maastrichtian (e.g., [72], [88], [94], [95], [96]) to early Paleocene (e.g., [68], [97], [98], [99], [100]). A correlation with latest Cretaceous Lancian age deposits of the northern Rocky Mountain region were bolstered with the report of the multituberculate mammal *Essonodon browni*, [86] and later by the report of the metatherian mammal *Glasbius* [87] from microvertebrate sites from the Naashoibito Member. Both mammals are otherwise known only from Lancian age sites of the northern Rocky Mountain region [36], [101] and are restricted to the upper part of the Hell Creek Formation (∼67–66 Ma) of Montana [80], [101].

Detrital sanidine grains recovered from a white sandstone facies above the base of the Naashoibito Member set a maximum depositional age of 66.3 Ma, consistent with a latest Cretaceous age for the Alamo Wash local fauna [37], [102]

Lehman [84] suggested that the Alamo Wash local fauna was Maastrichtian in age and part of an “*Alamosaurus* community” that occupied the Southwest near the end of the Cretaceous [84], [103].

## Results

### Quantitative tests

In the Systematic Palaeontology section below, we outline explicitly how the three lines of evidence (synapomorphies, PCA, DFAs) constrain the identifications and phylogenetic affinities of each morphotype. The PCA of all small theropod teeth returned five axes with the following eigenvalues and percentages of total variance explained by each axis: Axis 1 (3.22633, 64.527%), Axis 2 (0.904273, 18.085%), Axis 3 (0.53864, 10.773%), Axis 4 (0.215343, 4.3069%), Axis 5 (0.115418, 2.3084%). Coefficients for the five measurements on each axis are provided in the supplementary information. A morphospace depicting the positions of all small theropod teeth on the first two axes is presented in Figure 3A, and a simplified version showing only the positions of the San Juan Basin teeth is shown in Figure 3B.

SYSTEMATIC PALEONTOLOGY: TYRANNOSAUROID TEETH

Dinosauria Owen 1842 [104]

Theropoda Marsh, 1881 [105]

Coelurosauria von Huene, 1914 [106]

Tyrannosauroidea Osborn, 1905 [107]

#### Description

Here several small teeth from the Hosta Tongue, Point Lookout Sandstone, the De-na-zin Member, Kirtland Formation, and the Naashoibito Member are referred to Tryannosauroidea. All the teeth documented here lack roots and therefore likely represent shed teeth [46].

Carr and Williamson [108] reported a partial tooth (NMMNH P-27482) from the Hosta Tongue of the Point Lookout Sandstone (NMMNH locality L-297) that they referred to Tyrannosauridae. Here three additional teeth from the same locality are referred to Tyrannosauroidea. One tooth (NMMNH P-27483) is damaged and does not preserve denticles. Its referral to Tyrannosauroidea is based on size and robusticity. It is approximately 2.0 times longer mesiodistally than thick labiolingually (Appendix S1) and therefore more robust than teeth from the middle of the maxillary and dentary tooth rows of basal tyrannosauroids or *Alioramus*, but not as robust as in derived tyrannosauroids such as *Albertosaurus* or *Daspletosaurus* [109], [110]. Specimen NMMNH P-27484 (Fig. 4A–E) is a nearly complete tooth that is tall, labiolingually narrow, and recurved. It is about two times longer mesiodistally than thick labiolingually (Appendix S1). The mesial carina twists lingually towards the lingual surface of the tooth. The mesial carina and crown apex is worn, possibly through attritional wear and this has obliterated much of the details of the denticles on the mesial carina. Specimen NMMNH P27485 (Fig. 4F–K) has a lower CH than NMMNH P-27484, but is mesiodistally longer and more robust, with a mesiodistal length about 1.8 times the labiolingual thickness (Appendix S1). Both teeth have relatively small denticles (3.8–5 per millimieter; Appendix S1) that are smaller than those typically found in larger Campanian and Maastrichtian tyrannosauroid teeth (e.g., [108]; Appendix S1).

Several small teeth from the De-na-zin Member, Kirtland Formation are also referred to Tyrannosauroidea. Two shed teeth, NMMNH P-33903 (Fig. 4L–M), were surface collected after being found freshly eroded from a mudstone. They are interpreted to most likely represent teeth shed from a single individual, but they are not adjacent teeth but rather represent either different sides of the dentition, or upper and lower teeth from the same side of the dentition. Both teeth have tips that are rounded through attritional wear. Both teeth are relatively wide in cross section (the smaller of the two teeth has an FABL/BW of 1.66, which fulfills the definition of incrassate presented by Brusatte et al. 2010 and found only in derived tyrannosauroids), relatively coarsely denticulate, and ovoid in cross section. The mesial carinae deflect strongly to one side. A single tooth (NMMNH P-27280) is a complete shed tooth, found while excavating a partial hadrosaur skull. It is coarsely serrated (3.5/3.5 per mm) and is relatively narrow (FABL/BW = 2.29). It is nearly oval in cross section with a pronounced lingual-labial constriction at mid-length. Mesial and distal carinae are in-line with the long axis of the tooth. It is nearly bilaterally symmetrical in mesial (Fig. 4S) and distal views, but the mesial carina bends lingually. It exhibits subtle enamel wrinkles (see [111]) on the labial face of the crown, a feature commonly seen in tyrannosauroids and many other theropods.

Two small tyrannosauroid teeth are reported from the Naashoibito Member. NMMNH P-32819 is relatively crushed and distorted. The second tooth, NMMNH P-32567 (Fig. 4U–Y) is well preserved, but is missing a portion of its tip and base. It is labiolingually narrow (FABL/BW = 1.69, which is very near the cut-off distinguishing ziphodont from incrassate teeth by Brusatte et al. ([43]; Appendix S1) and coarsely serrated (4.5 per mm; Appendix S1), but with serrations finer than is typically seen in *Tyrannosaurus rex* [112].

#### Identification

Several teeth from the various samples are referred to Tyrannosauroidea on the basis of a number of features including crown shape and size and shape of denticles [18], [21], [40], [112]. The crowns of maxillary and dentary teeth of tyrannosauroids tend to be less recurved than in other theropods, with round to ovoid cross sections (incrassate morphology), and robust, wide, saddle- or chisel-shaped, widely spaced denticles [40] that are present on both the distal and mesial carinae (see [40]). One of these features, the incrassate tooth structure, has been recovered as a synapomorphy of Tyrannosauridae by phylogenetic analyses [43], [113]. Some or all of these features are seen in the teeth here classified as Tyrannosauroidea, which supports their referral to this group.

Several studies applying quantitative methods such as principal component analyses (PCA) of tooth shape and statistical analyses such as discriminant function analysis (DFA) concluded that it is difficult to distinguish the teeth of tyrannosauroid taxa as there tends to be considerable overlap in tooth morphology between adults of different species of tyrannosauroid [40]. Therefore, it is extremely difficult to identify isolated tyrannosaurid teeth to any level more finely than Tyrannosauroidea or Tyrannosauridae indet.

Another problematic issue in identifying tyrannosauroid teeth is ontogeny. Small tyrannosauroid teeth may represent early ontogenetic stages of adults. PCA of teeth representing several tyrannosaurid taxa and samples of what are thought to represent a population sample including juveniles of the tyrannosaurine *Albertosaurus sarcophagus* show that there are distinct morphological differences between the teeth of juvenile and adult tyrannosaurs [40], [46], [47] indicating that tyrannosauroids undergo significant allometric changes in tooth morphology through ontogeny [40], [46]. However, there is no independent way to age isolated teeth. Nevertheless, it may be most parsimonious to consider the small teeth to be those of early ontogenetic stages of adults [46] rather than potential distinct small-bodied taxa. Additionally, because juveniles of derived tyrannosauroid taxa do not possess the incrassate (proportionally labiolingually wide) teeth of adults, the lack of an incrassate morphology does not preclude referral to derived Tyrannosauroidea, but instead likely indicates a juvenile condition (e.g., [110], [114]).

#### Discussion of tyrannosauroid teeth

All the tyrannosauroid teeth reported here are small, with a FABL that is similar to those of the smallest tyrannosaur teeth reported by Currie et al. [21] from the Dinosaur Park Formation (7.2 mm) or by Buckley et al. [46] of *Albertosaurus sarcophagus* from the Barnum Brown *A. sarcophagus* bonebed of the upper Horseshoe Canyon Formation ([46]: supplementary information).

Two of the teeth tentatively referred to Tyrannosauroidea from the Hosta Tongue are smaller than the smallest teeth recovered from the Fruitland and Kirtland formations, with an FABL of 7.29 and 8.3 (Appendix S1). However, the Hosta Tongue tyrannosauroid teeth are significantly larger than any Late Cretaceous dromaeosaurid taxa and lack the distinctive denticles of other theropod taxa (e.g., Troodontidae, *Richardoestesia*, see below). Because of their small size and lack of discrete tyrannosauroid apomorphies such as an incrassate morphology, we only tentatively refer the Hosta Tongue teeth to Tyrannosauroidea here, based on their overall morphology and clear differences in size and shape from other Late Cretaceous theropod teeth.

If our identification of the Hosta Tongue teeth as tyrannosauroids is correct, they likely represent a different tyrannosauroid taxon than any previously described from North America. No diagnostic tyrannosauroid has been reported from North America from this age (∼85 Ma). Fragmentary skeletal remains of basal tyrannosauroids are present in the Upper Jurassic Morrison Formation [43], [115], [116], [117] and the tooth of a probable Early Cretaceous tyrannosauroid was reported from the Cloverly Formation of Wyoming [118]. In addition, isolated tyrannosauroid teeth have been reported from the Cenomanian Mussentuchit Member of the Cedar Mountain Formation [119], [120] and Dakota Formation , the Turonian – lower Campanian Straight Cliffs Formation [32], [33] of Utah, and the Santonian Milk River Formation of Alberta [23]. A putative tyrannosauroid has been reported from the Turonian age Moreno Hill Formation [121], [122], but this specimen has not yet been described. The oldest named derived tyrannosauroid ( = tyrannosaurid) taxon from North America is *Lythronax*, from rocks of ∼80 Ma in Utah [123], which is about five million years younger than the Hosta Tongue teeth.

The sample of “tyrannosaurine” teeth that Larson [23] described from the Santonian Milk River Formation is of an age similar to that of the Hosta Tongue and warrants further discussion. The Milk River sample is larger than that of the Hosta Tongue (number of lateral teeth = 28) and includes teeth smaller than any reported from the Hosta Tongue (the smallest has a FABL of 4.57 mm; [23]). These have a mean FABL of 11.79 [23] and a denticle density similar to that of the Hosta Tongue sample. The size range of the Milk River teeth encompasses the Hosta Tongue teeth and we are unable to find any significant morphological differences between the two samples, which is not surprising considering the difficulty of distinguishing teeth between other tyrannosauroid taxa. However, it is noteworthy that both the Hosta Tongue and Milk River samples contain teeth that are substantially smaller than those reported from larger samples representing derived tyrannosauroids collected from younger strata of the Western Interior.

Numerous tyrannosauroid teeth have previously been recovered from the Fruitland and Kirtland formations [108]. However, no diagnostic tyrannosauroids have been recovered from the De-na-zin Member [108], a unit that is similar in age to the underlying members of the Kirtland and Fruitland formation, where all diagnostic tyrannosauroid specimens, including the subadult specimen NMMNH P-25049, can be referred to a single taxon, *Bistahieversor sealeyi* [124]. Although it remains possible that more than one tyrannosauroid taxon is present in the late Campanian of the Fruitland and Kirtland formations, there is no evidence of an additional taxon and it is therefore most parsimonious to consider the small shed teeth from these units to be from individuals of early ontogenetic stages of *B. sealeyi*. Therefore the small teeth documented here give additional information on the tooth morphology of early ontogenetic stages of this taxon, and indicate that like in more derived tyrannosauroids (tyrannosaurids) the teeth of juveniles were mostly thinner and more delicate than the incrassate teeth of adults (e.g. [110], [114]). Magana et al. [125] reported that a principal components analysis of isolated teeth from the Kirtland Formation resulted in teeth being clustered in two groups, which they interpreted as separate groupings for adults and juveniles.

Several large tyrannosauroid teeth are also known from the Naashoibito Member [28], [108] and these have been tentatively referred to *Tyrannosaurus rex* [108], [126]. Carr and Williamson based this identification on the large size of the largest teeth and large size of the denticles, especially on large teeth. Tooth and denticle size of *T. rex* exceed those of all other tyrannosauroid taxa [108]. The largest reported Naashoibito Member tyrannosauroid teeth are similar in size to the largest reported *T. rex* teeth. The two teeth reported here, NMMNH P-32567 and 32819, are relatively small (Appendix S1) and similar in size to the smallest tyrannosauroid teeth reported from the Late Campanian. Although some workers argue that a “dwarf tyrannosaur” was present and lived sympatrically with *Tyrannosaurus rex* in the latest Cretaceous of western North America [127], the evidence to support this is not compelling. Instead it is more likely that small specimens referred to a “dwarf tyrannosaur” represent early ontogenetic stages of *T. rex* [114], [128]. Although no generically diagnostic cranial or postcranial bones have been recovered from the Naashoibito Member, no specimens contain features that would contradict a referral to *T. rex*. Moreover, diagnostic *T. rex* specimens are known from the Maastrichtian of central Utah [129], south-central New Mexico [108], [130], and West Texas [18]. Therefore, it is most parsimonious to refer all tyrannosauroid teeth from the Naashoibito Member to *T. rex*.

Jasinski et al. [28] illustrated and referred two teeth from the Naashoibito Member, State Museum of Pennsylvania (SMP) VP-2505 and SMP VP-2529, to Dromaeosauridae indet. We have not seen VP-2505, but based on features described and figured in the original publication we suggest that it is instead referable to Tyrannosauroidea, and probably represents a subadult. This suggestion is based on its size (reported to have a “total length” of 34 mm), which based on the illustration ([85]: fig. 9e–f) we interpret to be the CH measurement. This size would be expected for the tooth of a juvenile tyrannosauroid, but large for a dromaeosaurid. Additionally, the denticle count on the distal carina, “12–13 denticles per 5 mm,” is coarse and similar to that of a large tyrannosaurid such as *Tyrannosaurus rex*. We suggest, therefore, that this tooth represents a subadult tyrannosauroid.

Sankey [10] suggested that latest Maastrichtian small theropod faunas based on teeth were less diverse than those of the late Campanian because small, young *Tyrannosaurus rex* would have competed with other small theropods for prey in latest Cretaceous terrestrial ecosystems, and therefore would have excluded other species of small-bodied theropods from latest Maastrichtian ecosystems. We believe that this is unlikely because large-bodied tyrannosauroids were present in all latest Cretaceous terrestrial ecosystems of western North America, including those of the late Campanian, and all would presumably have passed through the same small size range early in their life histories [43], [131]. The presence of so many small tyrannosauroid teeth in the Campanian faunas of the San Juan Basin, which likely represent juveniles of large-bodied species, corroborate this view.

SYSTEMATIC PALEONTOLOGY: DROMAEOSAURID TEETH

Coelurosauria von Huene, 1914 [106]

Dromaeosauridae Matthew and Brown, 1922 [132]

#### Dromaeosauridae Morphotype A Description

This is similar to the “Saurornitholestinae” morphotype of Larson and Currie [17], but not the “?*Dromaeosaurus* Morphotype A” of Sankey et al. (2002). The teeth are laterally compressed and recurved, and lack a basal constriction. Denticles are labiolingually narrow and sharp, and project apically. Mesial denticles, where present, are smaller than distal denticles, and are usually less than half the size of the distal denticles [21]. The carina of mesial teeth are deflected lingually so that it is positioned lingual to the midline of the tooth, but it does not project lingually as in teeth typically referred to “*Dromaeosaurus*” (Dromaeosauridae morphotype B here).

**Figure 5:**
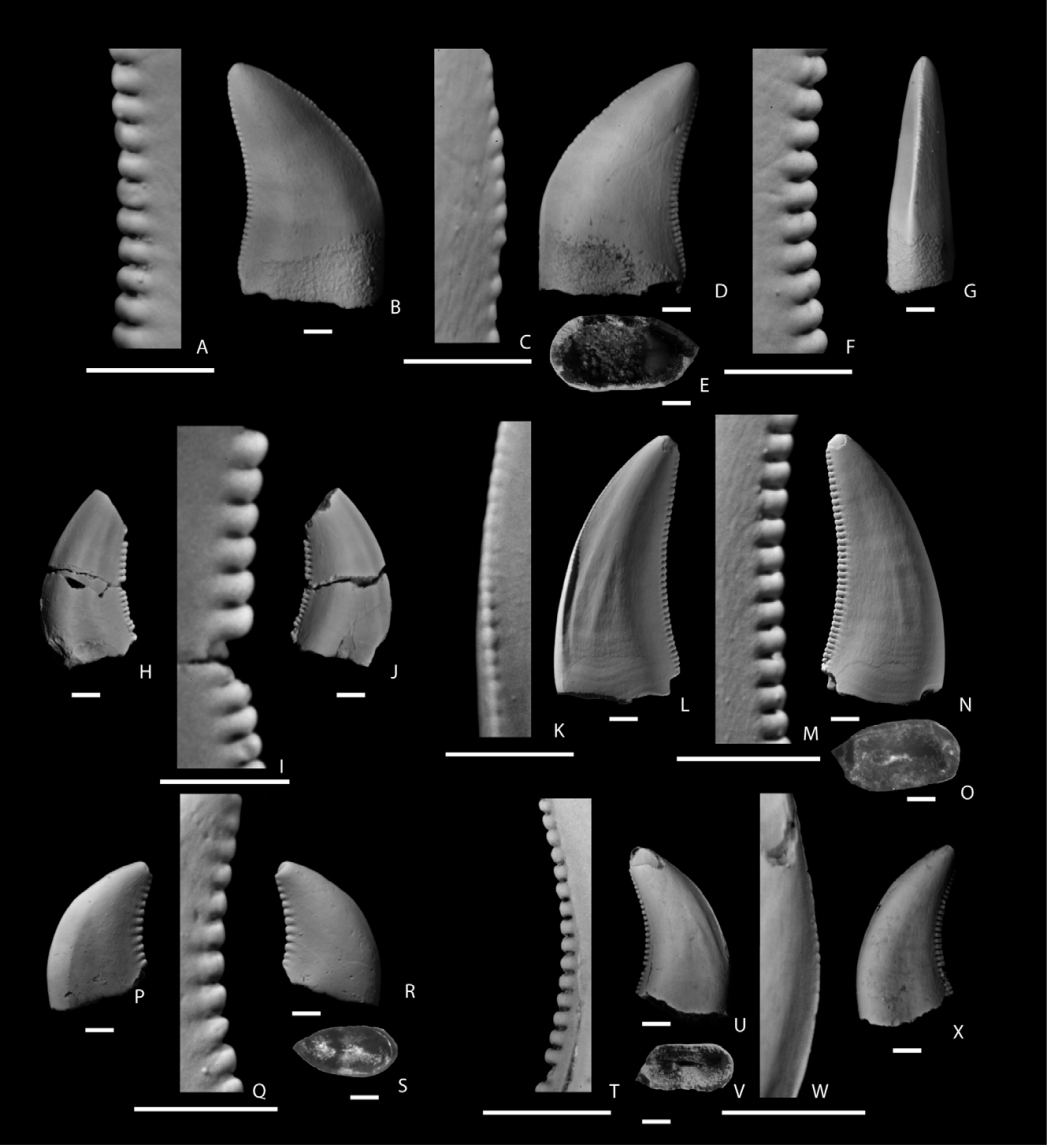
Dromaeosauridae Morphotype A. A–G, tooth of cf. Dromaeosauridae Morphotype A (NMMNH P-27481) from the Hosta Tongue, Point Lookout Sandstone showing lingual side of distal carina (A), lingual (B), lingual side mesial carina (C); labial (D), basal (E), labial side of distal carina (F), and mesial (G) views. H–J, tooth of Dromaeosauridae Morphotype A (NMMNH P-25054) from the Allison Member, Menefee Formation showing labial (H), labial view of distal carina (I), and lingual (J) views. K–O, tooth (NMMNH P-66896) from the Fossil Forest Member, Fruitland Formation showing lingual view of mesial carina (K), lingual (L), lingual view of distal carina (M), labial (N), and basal (O) views. P–S, tooth (NMMNH P-30003) from the Fossil Forest Member, Fruitland Formation showing lingual (P), lingual side of distal carina (Q), labial (R), and basal (S) views. T–X, tooth (NMMNH P-32814) from the Naashoibito Member, Kirtland Formation showing lingual side of distal carina (T), lingual (U), basal (V), lingual side of mesial carina (W), and labial (X) views. The scale bar below each image is 1 mm long.

A single tooth from the Hosta Tongue of the Point Lookout Sandstone, NMMNH P-27481, is very tentatively referred to Dromaeosauridae Morphotype A (Fig. 5A–G). It is ovoid in basal cross section, relatively narrow (FABL/BW = 1.97), and strongly recurved. The denticles are small (6 and 5 ADM and PDM, respectively), considerably smaller than the denticles of the teeth referred to Tyrannosauroidea indeterminate from the same locality (see above).

The denticles on the distal carina are approximately as labiolingually wide as proximodisally long. They are rounded and decrease in size towards the base of the crown. The denticles on the mesial carinae are smaller than those of the distal carina, but the disparity in size between the mesial and distal denticles is not as great as is typical in teeth of Dromaeosauridae Morphotype A from the San Juan Basin (Appendix S1) or the Dinosaur Park Formation [17]. The mesial denticles are worn and so their morphology is not distinct. The mesial carina is in-line with the long axis of the tooth, but the distal carina is closer to the lingual face of the tooth. The denticulation of the mesial carina begins about 1.5 mm above the base of the tooth. This tooth resembles what are interpreted to be distal maxillary or dentary teeth of Dromaeosauridae Morphotype A from the Fruitland and Kirtland formations (below), but it is significantly larger than teeth of that type. The specimen was included in the full PCA (Fig. 3) where it falls near the edge of a cluster of teeth from the Fruitland and lower Kirtland Formation referred to Dromaeosauridae Morphotype A and this forms the primary basis for our tentative referral to that morphotype.

Williamson [35] described a shed partial tooth from the early Campanian Menefee Formation and referred it to cf. *Saurornitholestes* sp. (NMMNH P-25054; Fig. 5H–J). It closely resembles teeth referred to *Saurornitholestes langstoni* from the Dinosaur Park Formation [21], [38]. The tooth is labiolingually narrow and recurved. The denticles on the distal carina, which were considered distinctive for the genus [21], [38], are labiolingually narrow and elongate, terminating in a hook that curves apically. Larson and Currie [17] described similarly-shaped denticles in Dinosaur Park Formation specimens as being “apically oriented” or being asymmetric with a shorter apical side, and used this feature as a qualitative character (character 1) and one of the defining characters of their “Saurornitholestinae” tooth morphotype. The mesial carina of P-25054 lacks denticles.

The most abundant small theropod teeth from the Fruitland and lower Kirtland (i.e., Hunter Wash, Farmington, and De-na-zin members) formations are referred to Dromaeosauridae Morphotype A (Fig. 5K–S). Approximately 30 percent lack mesial denticles. Several have a mesial carina that curves lingually, but the twist in the carina typically occurs at a point closer to the base of the tooth than the midpoint of the carina. Denticles are largest near the middle of the carina and decrease in size basally and apically as is typical for dromaeosaurids [24]. Denticles range from being rounded to asymmetrical with apically-hooked denticles. Teeth that are strongly recurved (see Fig. 5P–S) are presumed to be from a more posterior position in the tooth row [21] and these tend to have longer and more strongly apically-hooked distal denticles.

A single tooth from the Naashoibito Member, NMMNH P-32814 (Fig. 5T–X) is referred to Dromaeosauridae Morphotype A. It is similar to teeth referred to Dromaeosauridae Morphotype A from the Fruitland and lower Kirtland Formations (above). It is transversely compressed, recurved, and ovoid in basal cross section. The mesial carina is deflected mesially over its basal half. The mesial denticles are smaller than the distal denticles, but the largest mesial denticle is more than half the size of the largest distal denticle. The denticles are rounded as in *Acheroraptor* [24] rather than strongly apically-hooked as in *Saurornitholestes langstoni* [21].

Currie et al. [21] stated that one of the characteristic features of *Saurornitholestes* is the great disparity of size between mesial and distal denticles, with mesial denticles being usually less than half the size of the distal denticles. Larson and Currie [17] also consider the state of having mesial denticles much smaller than distal characters to be a defining qualitative character for their “Saurornitholestinae” tooth morphotype. For those specimens from the Fruitland and Kirtland formations with mesial denticles, there is a relatively large disparity in denticle size between mesial and distal carinae as in teeth referred to *Saurornitholestes* and *Acheroraptor.* Approximately two thirds of specimens from the Fruitland and lower Kirtland formations referred here to Dromaeosauridae Morphotype A possess mesial denticles. All of these have mesial denticals that are smaller than distal denticles, but in all cases, the largest mesial denticle of each tooth is more than half the size of the largest distal denticle.

Several teeth referred to Dromaeosauridae Morphotype A exhibit weak apicobasal ridges on the mesial half of their lingual and labial faces (see Fig. 5L, N), and on NMMNH P32814, they are present on the lingual face, but not the labial face, of the tooth (Fig. 5U, X). The ridges are similar to those described for *Acheroraptor* [24], but unlike the teeth of *Acheroraptor*, the ridges on the New Mexico specimens appear to be less pronounced and do not extend to the apical portion of the teeth.

#### Identification

Isolated teeth from the latest Cretaceous of North America attributed to dromaeosaurid dinosaurs have long been divided among just two taxa, *Dromaeosaurus* and *Saurornitholestes*, which were for many years the only two dromaeosaurid taxa known from the Late Cretaceous of North America that had an association between the dentition and diagnostic cranial bones [18], [19], [21], [24], [38]. These names have been applied to teeth from a wide geographic range across much of western North America and probably spanning several million years. More recently, however, Larson and Currie [17] applied various multivariate analyses to small theropod teeth from samples from many sites across North America ranging in age from Santonian through Maastrichtian, and found that samples of teeth referred to the same taxon from different locales could usually be distinguished quantitatively. This suggests that small theropods taxa likely had limited geographic ranges and showed considerable taxonomic heterogeneity over western North America through the Late Cretaceous. Assigning teeth from across the western interior to the specific genera *Dromaeosaurus* and *Saurornitholestes*, therefore, is not advisable and is not followed here.

A handful of phylogenetic characters can help assign isolated teeth, like the San Juan Basin specimens and much of the material described by Larson and Currie [17], to Dromaeosauridae and subclades. First, in their comprehensive phylogenetic analysis of dromaeosaurids, Turner et al. [16] found that Dromaeosauridae is united by a shared derived character of maxillary and dentary teeth that lack a basal constriction between the root and crown (character 88 in their analysis). This is also seen in some primitive coelurosaurs like tyrannosauroids and most toothed ornithomimosaurs, but a constriction is present in troodontids, most basal birds, toothed oviraptorosaurs, therizinosauroids, and most alvarezsauroids. The lack of constriction in the New Mexico Dromaeosaurid Morphotype A teeth, therefore, support their referral to Dromaeosauridae.

Second, Turner et al. [16] utilized a character regarding size differences between the mesial and distal denticles of individual teeth. They found that dromaeosaurids generally, except for *Dromaeosaurus albertensis*, have teeth in which the mesial denticles are substantially smaller than the distal denticles. Otherwise, among theropods that possess both mesial and distal denticles on their teeth, such proportionally small mesial denticles are only seen in a handful of primitive tyrannosauroids from the Middle Jurassic-Early Cretaceous [133]. The fact that the New Mexico teeth assigned to Dromaeosaurid Morphotype A have mesial denticles that are much smaller than the distal denticles means that they can be confidently referred to Dromaeosauridae based on the character optimization in Turner et al. [16].

Currie et al. [21] referred *Saurornitholestes langstoni* (and therefore the classic North American “*Saurornitholestes*” tooth morphotype) to the dromaeosaurid subclade Velociraptorinae, based on the assumption at the time that the nearly equally-sized mesial and distal denticles of *Dromaeosaurus* were representative of all dromaeosaurines and the proportionally smaller mesial denticles of *Velociraptor* and *Saurornitholestes* were representative of velociraptorines. Turner et al. [16], however, demonstrated that the equally-sized denticles of *Dromaeosaurus* are an aberration among dromaeosaurids and that the *Saurornitholestes*-like condition is widespread among dromaeosaurids (including in the dromaeosaurines *Atrociraptor* and *Achillobator*). Therefore, the presence of proportionally small mesial denticles cannot be used to assign the San Juan Basin teeth, or other isolated theropod teeth, to Velociraptorinae, but rather to the more inclusive group Dromaeosauridae.

Finally, Turner et al. [16] recovered “(all) maxillary and dentary teeth with serrations on both anterior and posterior margins” (character 83 in their analysis) to be a synapomorphy of Dromaeosaurinae, the restricted subclade of dromaeosaurids that includes *Dromaeosaurus*, *Utahraptor*, *Achillobator*, and *Atrociraptor*. This is a particularly homoplastic character among theropods, but one that is unusually seen among dromaeosaurids, as it is only scored for *Dromaeosaurus*, *Achillobator*, and *Atrociraptor*. In contrast, other dromaeosaurids like velociraptorines (e.g., *Velociraptor* and *Deinonychus*) and microraptorines (e.g., *Microraptor*) have some, but not all, teeth without serrations on the mesial carina. A handful of unusual dromaeosaurids, including the unenlagiines *Buitreraptor* and *Austroraptor* and the basal taxon *Mahakala*, lack denticles on all teeth. Based on the optimization of this character on the phylogeny of Turner et al. [16], Dromaeosauridae Morphotype A cannot be referred to Dromaeosaurinae or Unenlagiinae, but could represent a velociraptorine or another type of dromaeosaurid. This character also helps to understand why some, but not all, Dromaeosauridae Morphotype A teeth lack mesial denticles: because this feature is variable along the tooth row in individual taxa [21]. This dismisses potential criticism of lumping together teeth possessing and lacking mesial denticles within the same morphotype.

Additional evidence for the identifications of San Juan Basin Dromaeosaurid Morphotype A teeth comes from the PCA and DFAs. The tooth from the Menefee formation referred to cf. Dromaeosaurid Morphotype A and the teeth from the Fruitland and Kirtland formations, including the tooth from the Naashoibito Member, referred to Dromaeosauridae Morphotype A cluster together and broadly overlap with the distribution of all “Sauronitholestine” teeth compiled by Larson and Currie ([17], Fig. 4). When a DFA is performed to analyze the similarities between San Juan Basin Dromaeosauridae Morphotype A teeth and “Saurornitholestinae” morphotype teeth from the Dinosaur Park Formation, the hit ratio is 70.44% (79.43% if only the more complete specimens are included and 69.03% if only Fruitland-Kirtland specimens are compared to the Dinosaur Park Formation specimens). This is below the 90% threshold for recognizing a quantitative distinction between the two groups, which means that there is no clear evidence for their separation. Although this does not explicitly identify the San Juan Basin teeth as belonging to dromaeosaurids, or to a particular clade of dromaeosaurids, it is evidence that they belong to the same general group of theropods as the “Saurornitholestinae” teeth from the northern Rockies.

#### Discussion

Sullivan [29] tentatively referred an isolated tooth (SMP VP-1901) from the De-na-zin Member to *Saurornitholestes robustus*, a taxon based on an weathered and damaged frontal (SMP VP-1955). The specimen is laterally compressed, recurved and similar in general appearance to teeth from the Fruitland and lower Kirtland formations that we refer to Dormaeosauridae morphotype A. However, it is larger than any dromaeosaurid teeth that we document, with a reported fore-aft basal length of 6.5 mm. We have not examined the specimen directly and are unable to confirm that it is a dromaeosaurid tooth based on the published images [29: fig. 2]. Regardless, Turner [16] considered *S. robustus* to be a nomen dubium, arguing that the holotype of *S. robustus* is too damaged to show that it possesses synapormophies of *Saurornitholestes* or even Dromaeosauridae.

#### Dromaeosauridae Morphotype B Description

This is similar to the “Dromaeosaurinae” morphotype of Larson and Currie [17]. The teeth are laterally compressed and recurved without a basal constriction. The denticles on the mesial and distal carinae are subequal in size and rounded in lateral view.

Only a single tooth, NMMNH P-33148, from the Hunter Wash Member, Kirtland Formation, corresponds to teeth that Currie et al. [21] referred to *Dromaeosaurus albertensis*, but because it is not clear that isolated teeth are diagnostic to genus or species, it is referred to Dromaeosauridae Morphotype B. A second tooth, NMMNH P-30225 is incomplete, but has the distinctive lingually-projecting mesial carina of this morphotype. As in the teeth of *Dromaeosaurus albertensis*, the mesial and distal carinae are both positioned lingually, giving the tooth a basal cross section that is D-shaped, but asymmetrical. The denticles are small and chisel-shaped and subequal in size on the mesial and distal carinae.

#### Identification

Currie [134] considered Dromaeosaurinae to be taxonomically equivalent to the species-level taxon *Dromaeosaurus albertensis*, which at that time he considered to be the only clear member of the subfamily-level group. He listed the lingual twist of the mesial carina as one of the diagnostic character for the clade [24], [135]. However, subsequent phylogenetic analyses did not always recover a monophyletic Dromaeosaurinae and Velociraptorinae (see [16]), making it uncertain which tooth features may be unique to Dromaeosaurinae, assuming such a clade even exists. Turner et al. [16] conducted the most comprehensive phylogenetic analysis of Dromaeosauridae to date and recovered Dromaeosaurinae and Velociraptorinae as distinct clades, which are sister taxa among derived dromaeosaurids. Based on their analysis, Dromaeosaurinae is a stem-based clade that includes all dromaeosaurids more closely related to *Dromaeosaurus* than to *Velociraptor*, *Microraptor*, *Unenlagia*, and Avialae. Membership in this clade is limited, however, as it only includes *Dromaeosaurus*, *Utahraptor*, *Achillobator*, and *Atrociraptor*.

Three important phylogenetic characters of Turner et al. [16] are relevant to identifying teeth as belonging to Dromaeosaurinae. First, as outlined above, dromaeosaurines are unusual among dromaeosaurids in having mesial and distal serrations on all teeth. The tooth NMMNH P-33148 does have serrations on both carinae, but because this is only an isolated tooth it cannot be scored confidently for this character, which depends on having a complete or nearly complete dentition to ascertain whether either all or some teeth possess mesial denticles. Secondly, Turner et al. [16] found that the twisting mesial carina, noted by Currie [134] to be an unusual feature of *Dromaeosaurus albertensis*, is not present in any other dromaeosaurids, including close relatives of *Dromaeosaurus* like *Achillobator* and *Atrociraptor*. Therefore, possession of this twisting carina is a strong indicator that an isolated tooth belongs either to *Dromaeosaurus* or a dromaeosaurine that is more closely related to *Dromaeosaurus* than to any other taxon. Because the San Juan Basin Dromaeosaurid Morphotype B teeth possess this feature, they can be confidently identified as pertaining to such a dromaeosaurine. Third, as noted above, *Dromaeosaurus* is unique among dromaeosaurines in possessing mesial and distal denticles of approximately the same size, a feature also seen in the San Juan Basin Morphotype B tooth that further supports its identification as a *Dromaeosaurus*-like dromaeosaurine.

**Figure 6:**
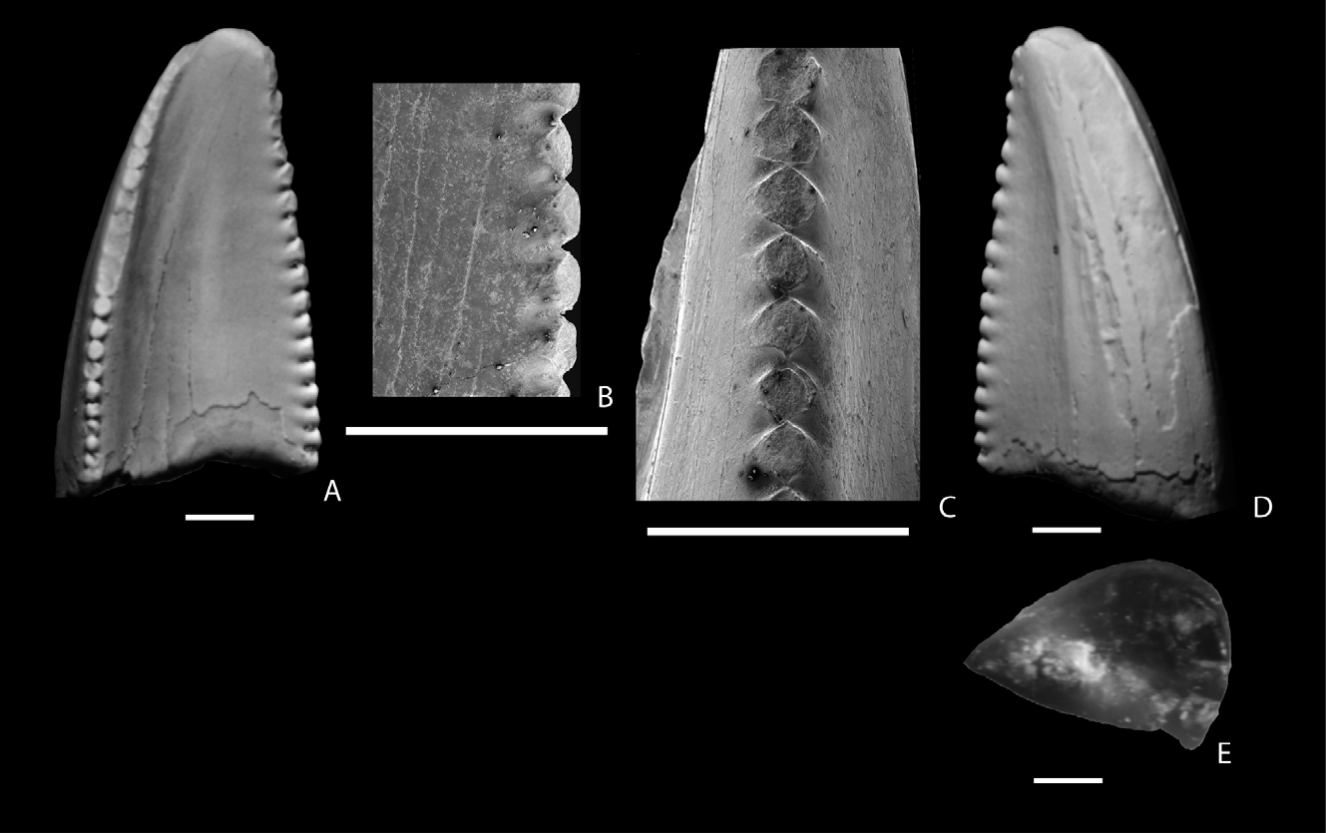
Dromaeosauridae Morphotype B (NMMNH P-33148) from the Hunter Wash Member, Kirtland Formation showing lingual (A), lingual side of distal carina (B), distal side of distal carina (C), labial (D), and basal (E) views. The scale bar below each image is 1

The tooth NMMNH P-33148 (Fig. 6) closely resembles those of *Dromaeosaurus albertenesis* and teeth from the Dinosaur Park referred to that taxon [21], [38]. Larson and Currie [17] show an additional “Dromaeosaurine” morphotype in the Dinosaur Park fauna which they identify as *Zapsalis abradens*. It possesses pronounced apicobasal striations on the lingual and labial faces of the crown. It lacks a lingually twisting mesial carina possessing instead a pronounced mesially projecting blade-like mesial carina, sometimes bearing small denticles [17], [136]. There are no *Zapsalis abradens-*like teeth in the San Juan Basin sample.

Some equivocal evidence for the identifications of San Juan Basin Dromaeosaurid Morphotype B teeth comes from the PCA. The sole relatively complete tooth that is referred to Dromaeosaurid Morphotype B plots outside of the distribution of “Dromaeosaurine” teeth compiled by Larson and Currie [17] and is at the margin of the plots of all the small theropod teeth that they compiled. This appears to be related to the relatively small size of the tooth (Appendix S1, S2), which is close in size to the smallest reported for ”Dromaeosaurinae” from the Dinosaur Park Formation [17], as well as the relatively large size of denticles. However, the size of the denticles is not larger than is found in the larger isolated teeth referred to *Dromaeosaurus albertensis* from the Dinosaur Park Formation [17]. This is perhaps suggestive of the San Juan Basin Dromaeosauridae Morphotype B representing a distinct taxon, with a combination of small crown size and large denticles, although this is very difficult to conclusively test with such small sample sizes. Unfortunately the sample size of San Juan Basin Dromaeosauridae Morphotype B teeth is also too small for a conclusive DFA comparing it to “Dromaeosaurinae” teeth from the northern Rockies.

Troodontidae Gilmore 1924 [137]

Troodontidae genus and species indeterminate

#### Description

Troodontids, including the San Juan Basin specimens, are characterized by sharp, recurved teeth with distinctive denticles that are relatively large, tapering, and hook-shaped, projecting towards the tip of the tooth. Most teeth referred to Troodontidae are bulbous near their base with a nearly circular basal cross section and a pronounced basal constriction [21], [38].

**Figure 7:**
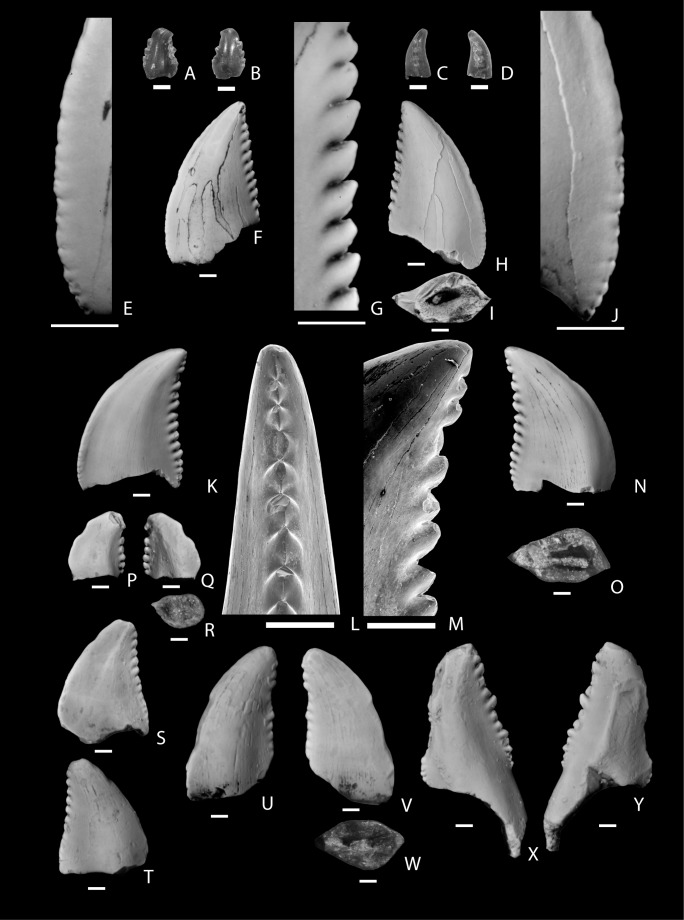
Troodontidae genus and species indeterminate. A–D, tooth (KUVP 96932) from the Fossil Forest Member, Fruitland Formation showing labial (A), lingual (B), mesial (C), and distal (D) views; E–J, tooth (NMMNH P-68395) from the De-na-zin Member, Kirtland Formation showing labial side of mesial carina (E), labial (F), labial side of distal carina (G), labial (H), basal (I), and labial side of mesial carina (J) views; K–O, tooth (NMMNH P-32772) from the Naashoibito Member, Kirtland Formation showing lingual (K), distal (L), lingual (M), lingual side of distal carina, labial (N), and basal (O) views; P–R, tooth (NMMNH P-33521) from the Naashoibito Member, Kirtland Formation showing lingual (P), labial (Q), and basal (R) views; S–T, tooth (NMMNH P-33520) from the Naashoibito Member, Kirtland Formation showing labial (S) and lingual (T) views; U–W, tooth (NMMNH P-22566) from the Naashoibito Member, Kirtland Formation showing labial (U), lingual (V), and basal (W) views; X–Y, tooth (NMMNH P-33901) from the Naashoibito Member, Kirtland Formation showing labial (X) and lingual (Y) views. The scale bar below each image is 1 mm long.

Hall [26] referred numerous teeth from the Fossil Forest Member of the Fruitland Formation to Troodontidae, but only one of these, KUVP 96932 (Fig. 7A–D), is here regarded as a troodontid. The other teeth are referred to Dromaeosauridae Morphotype A. KUVP 96932 is a small tooth with a bulbous base and relatively few and large denticles on both the mesial and distal carinae. It appears nearly symmetrical in lateral profile except for the extreme tip which curves abruptly distally. The tooth is curved in mesial and distal views so that the lingual surface is concave and the labial surface is convex. Only four denticles are present on the mesial carina and they steadily increase in size from the base of the crown. Many of the denticles are missing on the distal carina, but based on the preserved denticle bases, only four were present on the distal carina as well. They also appear to have increased in size towards the tooth tip. Each denticle is hooked and projects towards the tip of the tooth.

Lehman [84] originally identified an isolated tooth (University of New Mexico FKK-014) from the Naashoibito Member as “Saurornithoididae”, a taxon that has subsequently been synonymized with Troodontidae [134], [135]. This specimen (now NMMNH P-22566; Fig. 7S–T) is here referred to Troodontidae genus and species indeterminate. Williamson [138] and Williamson and Weil [30] have mentioned the presence of “*Troodon*” in the Alamo Wash local fauna from the Naashoibito Member. An additional isolated tooth of an “indeterminate troodontid” (SMP VP-3341) was illustrated and described by Jasinski et al. ([28]: fig. 9g–h).

The Naashoibito Member troodontid teeth are recurved with expanded bases and a pronounced basal constriction. Most specimens are poorly preserved or show abrasion that has removed fine surface detail, but one tooth (NMMNH P-32772, Fig. 7K–O) is exceptionally preserved, although attritional wear has removed details of the mesial carina including the tips of many of the denticles. It clearly exhibits the large, hook-shaped tapering, apically-pointing denticles on the distal carina that are characteristic of troodontids. The denticles on the distal carina decrease in size near the base of the crown. The mesial carina is lingually placed and projects mesiolingually. While most of the denticles on the mesial carina have been obliterated through attritional wear, several denticles are at least partially preserved near the base of the tooth. These are smaller than denticles on the distal carina. The base of the tooth is incompletely preserved, but it is sufficient to show a basal constriction.

Troodontid teeth show considerable variety of morphology according to their position in the jaw [21], [139] and this most likely explains the large range of variation in teeth referred to Troodontidae from the Naashoibito Member. One tooth, NMMNH P-33521 (Fig. 7P–Q), is small and possibly possesses a large mesial carina, although this is damaged through abrasion, and it may represent a tooth from a mesial position of the dentition. Otherwise, it is difficult to explicitly pin down where individual teeth may have fit in the jaws.

#### Identification

The New Mexico teeth possess important phylogenetic characters of troodontids that allow them to be confidentially assigned to this unusual group of bird-like theropods. Following the analysis of Turner et al. [16], the teeth possess two synapomorphies of Troodontidae or internal subclades. First, the serrations are hooked towards the tip of the crown as in *Troodon*, *Zanabazar*, and *Saurornithoides*, unlike the simple serrations that project essentially perpendicular to the crown in most other theropods (character 87 in their analysis). Second, the serrations are enormous, with only 2–3 coarse serrations per mm. This condition is also seen in *Troodon*, *Zanabazar*, *Saurornithoides*, and *Sinornithoides*, but differs from the much smaller serrations of almost all other theropods (usually 5+ serrations per mm in all but the smallest teeth) (character 86 in their analysis).

Additional evidence for the identification of San Juan Basin Troodontidae teeth comes from the PCA and DFAs. The single troodontid tooth from the De-na-zin Member, Kirtland Formation, falls well inside the cluster of Dinosaur Park Formation troodontid teeth compiled by Larson and Currie ([17], Fig. 3). The three troodontid teeth from the Naashoibito Member plot near the margin of the cluster of all troodontid teeth from the northern Rockies in the Larson and Currie dataset ([17], Fig. 3). When a DFA is performed to analyze the similarities between the Naashoibito Troodontidae teeth and “Troodontidae” morphotype teeth from the Dinosaur Park Formation, the hit ratio is 76.6% (83.72% if only the more complete specimens are included). This is below the 90% threshold for recognizing a quantitative distinction between the two groups, which means that there is no clear evidence for their separation. A similar hit ratio of 65.91% (75% if only the more complete specmiens are included) was found when a DFA is performed to analyze the similarities between the Naashoibito Troodontidae teeth and “Troodontdiae” morphotype teeth from the Horseshoe Canyon Formation. Although this does not explicitly identify the San Juan Basin teeth as belonging to troodontids, it is evidence that they belong to the same general group of theropods as the “Troodontidae” teeth from the northern Rockies.

#### Discussion

The single troodontid tooth reported from the Fruitland Formation, KUVP 96932, is atypical compared to teeth of troodontids reported from other Late Cretaceous North American locales in having a small size, and in the nearly symmetrical, conical profile view, with a sharply posteriorly projecting tip. It possibly represents an early ontogenetic stage of a *Troodon*-like taxon, although this is difficult to test given the relatively small samples of troodontid teeth available for comparison and poor understanding of troodontid ontogeny.

The single tooth from the De-na-zin Member more closely resembles a “typical” troodontid tooth similar to those referred to *Troodon formosus* reported from the Dinosaur Park Formation of Alberta (e.g., [21], [38]) or troodontid teeth reported from the upper Campanian Kaiparowits Formation of Utah [32], [140]. They do not resemble the teeth of *Pectinodon abradens*, a taxon from the upper Maastrichtian Lance and Hell Creek formations of Wyoming, Montana, and nearby states that is based on distinctive isolated teeth that has been referred to Troodontidae [20] which lack a basal constriction and have denticles restricted to the distal carina.

Some of the New Mexico teeth exhibit a large difference between the sizes of mesial and distal denticles. These differences are relatively large compared to troodontid specimens of described from the Dinosaur Park Formation the Lance and Hell Creek Formations of Montana [10], and potentially could represent a taxonomically distinctive character of the New Mexico specimens. This observation led us to perform two statistical tests to assess the differences between the Naashoibito troodontids from the San Juan Basin and those from the Dinosaur Park Formation. First, a DFA reports a hit ratio of 94.23% (100% if only the more complete specimens are included), which is above the 90% threshold for recognizing a clear quantitative difference between two samples. Second, a two-groups permutation test based on PC scores recovers a p value of less than 0.0005 (both when all teeth are analyzed and only the more complete specimens are included), indicating a statistically significant difference in the means of the two groups in morphospace. Both tests indicate that the San Juan Basin troodontids are quantitatively distinct from their northern counterparts. We caution, however, that these results may be driven by the small sample size of the San Juan Basin troodontids, and must be reassessed as new specimens increase the available sample.

The Naashoibito troodontid teeth overlap in size with those reported from both the late Campanian Dinosaur Park Formation (FABL range of 2 to 9.8 mm) and the lower Maastrichtian Prince Creek Formation of Alaska (FABL range of 5.4 to 14.3 mm) as reported in Fiorillo ([141]: table 2). Although the sample size is small and many of the Naashoibito teeth are too incomplete to get a precise measurement, most are clearly larger than the mean FABL of 4.96 mm for the Dinosaur Park troodontid teeth determined by Fiorillo ([141]: table 2) and of the small sample (FABL = 2.77; n = 3) measured by Sankey et al. ([38]: appendix 1.7) as well as the mean FABL of 2.47 mm of troodontid teeth (“*Troodon*” sp. and “*Troodon* sp. Flat Morphology”) from the Hell Creek Formation ([10]: table 8.2). However they are also substantially smaller than the largest teeth reported from Alaska, with a mean FABL of 9.78 ([141]: table 2), and “Troodon sp. Large Morphology” from the Hell Creek Formation [10]. The latter is reported to be of similar size to those of Alaska (Sankey, 2008) evidently based on a single tooth, University of California, Museum of Paleontology (UCMP) 186979. Sankey [10] did not provide measurements of this tooth, but based on scaled images ([10]: fig. 8.3,13–16) it exceeds the size of any Naashoibito troodontid.

The sample of small theropod teeth from the Naashoibito Member is small, but troodontid teeth make up a large portion of the total sample that has been collected to date (Appendix S1). Naashoibito troodontids comprise a similar large proportion of the small theropod teeth as those of the lower Maastrichtian Prince Creek Formation fauna of Alaska [141].

SYSTEMATIC PALEONTOLOGY: THEROPOD TEETH INCERTAE SEDIS

Family Incertae sedis

*Richardoestesia* Currie, Rigby, and Sloan, 1990 [21]

#### Background

The genus *Richardoestesia* was erected by Currie et al. [21] based on the type of *R. gilmorei*, which consists of a pair of partial dentaries that contain several unerupted and germ teeth from the upper Campanian Dinosaur Park Formation of southern Alberta. Teeth of *Richardoestesia* are bladelike with small, rounded denticles. Mesial denticles are similar in size and shape to distal denticles, or absent. Sankey [18] erected a second species, *R. isosceles* based on an isolated tooth from the upper Campanian Aguja Formation, West Texas, that possessed a less recurved and more nearly erect profile.

Longrich [20] argued that previous interpretations that these teeth represent distinct taxa are incorrect and that both can be referred to a single taxon with a heterodont dentition. [20] further proposed that many teeth referred to *Paronychodon* represent the mesial dentition of this taxon.

Cf. *Richardoestesia* sp.

#### Description

**Figure 8:**
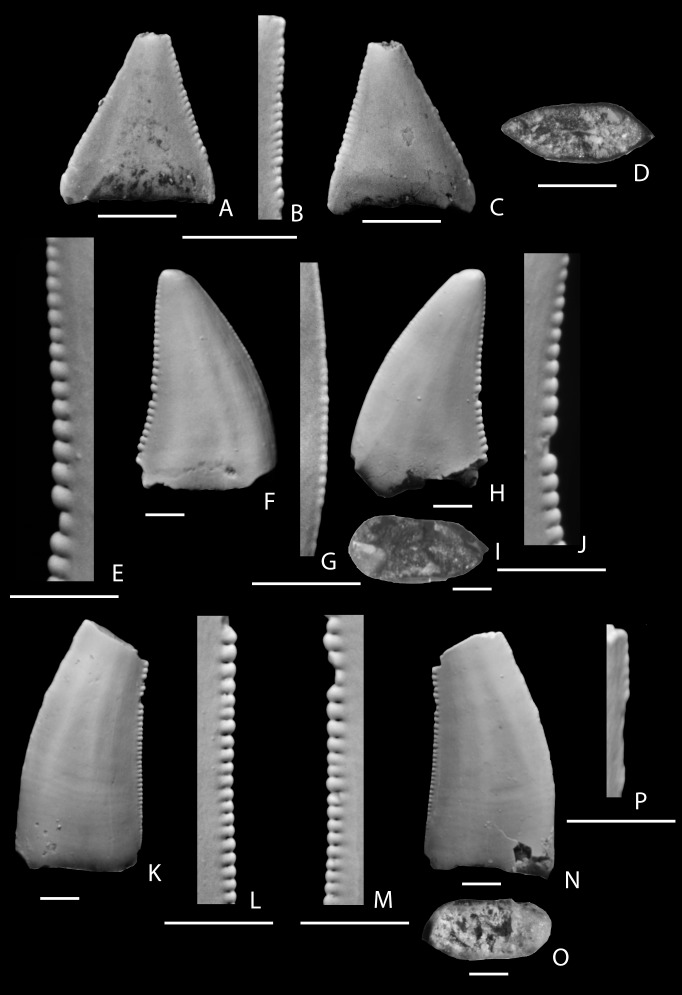
Cf. *Richardoestesia* spp. from the San Juan Basin, New Mexico. A–D, cf. *Richardoestesia* sp. tooth (NMMNH P-52503) from the Fruitland Formation showing labial (A), labial side of distal carina (B), lingual (C), and basal (D) views; E–J, cf. *R. gilmorei* tooth (NMMNH P-33482) showing lingual side of distal carina (E), lingual (F), lingual side of mesial carnia (G), labial (H), basal (I) and lingual side of distal carina (J) views; K–P, tooth (NMMNH P-32753) showing labial (K), labial side of distal carina, (L), lingual side of distal carina (M), lingual (N), basal (O), lingual side of mesial carina (P) views. The scale bar below each image is 1 mm long.

A small tooth, NMMNH P-52503 (Fig. 8A–D) from the Fossil Forest Member, Fruitland Formation, is similar to *Richardoestesia isosceles*, in that it is laterally compressed, with a suboval basal cross section and a nearly erect profile with only a slight distal cant. However, it differs from *R. isosceles* in having more steeply converging mesial and distal carinae and very small (PDM = 12), rounded denticles on the distal carina. The tooth is slightly constricted at the crown-root juncture by rounding at the base of the mesial and distal carinae. We suggest that this tooth may represent an early ontogenetic stage of *Richardoestesia* sp.

Other teeth from the Friutland and Kirtland formations that we refer to cf. *Richardoestesia* sp. are larger than NMMNH P-52503, laterally compressed, and recurved to some extent, with minute, rounded denticles. Most specimens referred to this taxon in the San Juan Basin sample lack denticles on the mesial carina, but when present, they are typically smaller than denticles on the distal carina. There are typically 9–15 denticles per mm on the mesial carina and 6–9 denticles per mm on the distal carina (Appendix S1). Denticles decrease in size apically. Subtle apicobasal ridges may be present on both the lingual and labial sides of the crown.

#### Identification

The tooth tentatively referred to *Richardoestesia* sp. (NMMNH P-52503) has small, rounded denticles on the distal carina and the mesial and distal margins are straight rather than curved as in other small theropod taxa. The tooth plots far outside the envelope of *Richardoestesia* and all theropod teeth compiled by Larson and Currie [17](Fig. 3). The other teeth from the San Juan Basin tentatively referred to *Richardoestesia* plot largely outside of the cluster of teeth of *R. gilmorei* and *R. isosceles* compiled by Larson and Currie [17], although there is a small area of overlap between these samples. All specimens that preserve mesial and distal denticles show markedly smaller mesial denticles. This differs from specimens previously referred to either *R. gilmorei* or *R. isosceles*, and is likely a major reason why the San Juan Basin teeth (despite their general resemblance to *Richardoestesia* and possession of characteristic features of the morphotype) fall outside of the PCA cluster of other *Richardoestesia* teeth from more northern regions.

#### Discussion

NMMNH P-52503 does not easily fit into either the *Richardoestesia gilmorei* or *R. isosceles* morphotypes. We suggest that NMMNH P-52503 represents an early ontogenetic stage of a *Richardoestesia* species due to its very small size and tiny distal denticles.

The San Juan Basin teeth tentatively assigned to *Richardoestesia* differ from other samples of *Richardoestesia* from the latest Cretaceous of North America in that the mesial denticles, when present, are substantially smaller than the denticles on the distal carina. In contrast, other *Richardoestesia* teeth have mesial and distal denticles that are nearly equal in size. Because the San Juan Basin sample falls outside of either morphotype as defined by Larson and Currie [17], we only tentatively refer these specimens to *Richardoestesia* sp. Moreover, we find that Longrich's [20] argument that *Richardoestesia gilmorei* and *R. isosceles* represent one taxon is supported by two observations of the sample of cf. *Richardoestesia* sp. teeth from the Fruitland Formation and Hunter Wash and De-na-zin members, Kirtland Formation. First, there appears to be a continuum of morphology between the highly erect and symmetrical teeth that closely resemble those referred to *R. isosceles* (mesial teeth, sensu Longrich, [20]) and the more recurved teeth (distal teeth, sensu Longrich [20]) tentatively referred to *R. gilmorei*. Second, because of the distinctive character of mesial denticles smaller than distal denticles is shared between both *Richardoestesia* tooth morphologies from the San Juan Basin (see Figure 8E–J and 8K–P), it is likely that they represent different tooth positions from a single heterodont taxon that is characterized by an autapomorphic morphology of proportionally tiny mesial denticles. Otherwise, to maintain separate taxa for the two morphotypes, one would need to argue that two distinct species both possess an identical derived feature (evolved independently) that is not seen in large samples of other representatives of those two distinct species from more northern regions.

The small sample of cf. *Richardoestesia* teeth from the Naashoibito Member contains only one specimen (NMMNH P-46389) that preserves both mesial and distal denticles. Those denticles are small and rounded and the denticles of the mesial and distal carinae are subequal in size. This tooth, therefore, is similar to either *R. gilmorei* or *R. isosceles* based on denticle characters. However, we are unable to assign it to either species and consider referral to the genus tentative because of remaining questions regarding the validity of the taxon [20].

Teeth referred to *Richardoestesia gilmorei*, cf. *R. gilmorei*, *R. isosceles*, or cf. *R. isosceles* have remarkably long stratigraphic and geographic ranges in Late Cretaceous deposits of western North America, with a temporal range of Santonian through Maastrichtian and a geographic range extending from West Texas to southern Alberta (e.g., [17], [18], [23], [38]) and it therefore is likely that these represent more than one taxon. The late Campanian sample tentatively referred to *Richardoestesia* may represent a new taxon with heterodont dentition that includes both *gilmorei*-type and *isosceles*-type teeth. In some local faunas *Richardoestesia* or cf. *Richardoestesia* sp. is incredibly abundant, including in the late Campanian microvertebrate faunas of the Fruitland and Kirtland Formations.

The Fruitland and lower Kirtland formation sample of cf. *Richardoestesia* sp. contains teeth that are relatively mesiodistally short, but which have a pronounced bend near their base so that the apex of the tooth points distally. These teeth are all fragmentary, but they resemble a tooth (Field Museum of Natural History PR 2899) that Gates et al. [136] described as representing a morphology not previously described in the literature to their knowledge. We suggest that this tooth represents cf. *Richardoestesia* sp.

*Paronychodon* Cope 1876 [142]

#### Background

The name *Paronychodon* is often applied to unusual theropod teeth from Late Cretaceous North America faunas. “Typical” *Paronychodon* teeth lack denticles, are laterally compressed with flattened lingual faces, and have marked apicobasal striations. Some are described as having a basal constriction [21]. It has been suggested that teeth with the classic *Paronychodon* morphology are abnormally developed teeth of theropods with a more traditional dromaeosaurid-like dentition, and therefore not representative of a unique taxon [21]. More recently Larson and Currie [17] suggested that *Paronychodon lacustris* may be a valid taxon, although they did not include these teeth in their analysis and thus did not quantitatively test whether they possess a distinctive morphology relative to other Late Cretaceous small theropod teeth. On the other hand, Longrich [20] suggested that *Paronychodon* teeth may be neither pathological nor representative of a distinct taxon, but rather represent the mesial dentition of species of *Richardoestesia*. Determining which of these many hypotheses is correct is an issue that has long befuddled researchers, and is far outside of the scope of this paper. Resolution will probably only come with the discovery of complete or near-complete in situ *Paronychodon* dentitions.

#### Description

**Figure 9:**
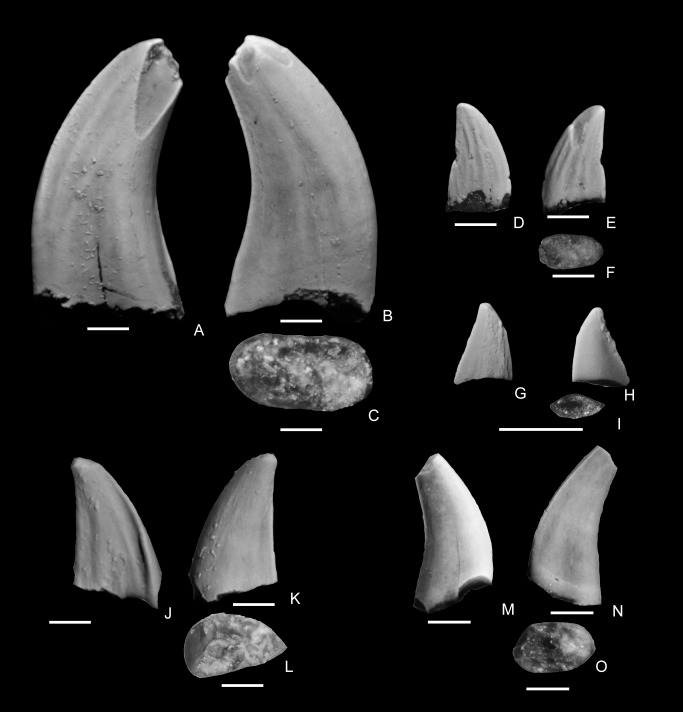
“*Paronychodon*” and unidentified theropod teeth. A–C, tooth of “*Paronychodon*” (NMMNH P-30233) showing lingual (A), labial (B), and basal (C) views; D–F, tooth of “*Paronychodon*” (NMMNH P-30218), showing lingual (D), labial (E), and basal (F) views; G–I, tooth of unidentified theropod (NMMNH P-30276) showing lingual (G), labial (H), and basal (I) views; J–L, tooth of unidentified theropod (NMMNH P-53360) showing lingual (J), labial (K), and basal (L) views; M–O, tooth of unidentified theropod (NMMNH P-38424) showing labial (M), lingual (N), and basal (O) views. The scale bar below each image is 1 mm long.

Among the Fruitland and lower Kirtland formation sample, two *Paronychodon* morphotypes can be recognized. The first *Paronychodon* morphotype is represented by NMMNH P-30233 (Fig. 9A–C). It is a laterally compressed tooth with an ovoid cross section. It is recurved and possesses sharp mesial and distal carinae, but lacks denticles. The lingual face of the tooth is nearly flat with pronounced apicobasal ridges. The second morphotype, represented by NMMNH P-30218 (Fig. 9D–F), is smaller than teeth of the first morphotype. It has an ovoid base and is laterally recurved, although less so than teeth of the first morphotype such as NMMNH P-30233. Additionally, compared to the first morphotype it lacks a flattened side and bears much more pronounced apicobasal ridges on both the lingual and lateral faces of the tooth crown.

#### Discussion

Both San Juan Basin morphotypes are dissimilar to both *Paronychodon lacustris* morphotypes described from the Campanian Judith River Group of Alberta by Sankey et al. [38] in having less pronounced apicobasal ridges and in lacking ridges that anastomose from the apex to the base of the crown. The second *Paronychodon* morphotype resembles teeth of *Zapsalis abradens* [17]. It is laterally compressed and recurved with strong apicobasal ridges and a nearly straight distal carina. However, it differs from *Zapsalis abradens* in lacking denticles on the distal carina. No teeth in the San Juan Basin sample with pronounced apicobasal ridges also possess denticles on the distal carina and therefore none can be referred to the *Zapsalis abradens* morphotype.

## Systematic Paleontology: Theropod Teeth Unidentified

### Theropoda Unidentified

#### Description

A number of small teeth, exhibiting a range of morphologies, cannot be easily assigned to any major theropod tooth morphotypes based on discrete characters. All are small, and lack denticles and the pronounced continuous apicobasal ridges that are typically found in *Paronychodon*. One specimen, NMMNH P-30276 (Fig. 9G–I), is very small, blade-shaped, triangular tooth, with rugose enamel that forms irregular ridges that run apicobasally. Another tooth, NMMNH P-53360 (Fig. 9J–L), has a nearly D-shaped cross section with several weak apicobasal ridges on the flat lingual face. A third puzzling tooth, NMMNH P-38424 (Fig. 9M–O), is ovoid in cross section, only slightly laterally compressed, strongly recurved, and has weak mesial and distal carinae. It does not appear to have a constriction at the base.

#### Discussion

All the unidentified theropod teeth are relatively small and may represent early ontogenetic stages of one or more small theropod taxa. If correct, this suggests that one or more small theropods may undergo allometric changes in tooth morphology through ontogeny which would result in a broader range of tooth variability in some small Late Cretaceous theropod taxa than is currently recognized. This, in turn, may make it difficult to distinguish ontogenetic and taxonomic signals in multivariate statistical analyses such as those performed by Larson and Currie [17] and the PC analyses performed here. Distinct clusters of teeth in morphospace could, in some instances, reflect ontogenetic differences rather than taxonomic differences. It is difficult to account for ontogenetic effects in such multivariate analyses, because it is usually very difficult to determine a priori whether individual teeth represent juveniles or adults. Larson and Currie ([17]: p. 13) did not account for ontogeny in their analyses, but noted that “some categories (of teeth) may be different ontogenetic stages of a single species separated by size alone, (but) differences in denticle morphology usually preclude such arguments.” Note that this statement referred to denticle shape, not presence/absence. The assumption implicit in this statement is that theropods do not change their denticle morphology (size and shape) during ontogeny. This assumption may or may not be correct for small theropods like dromaeosaurids and troodontids, because ontogenetic growth series of individual taxa are not available for assessing how dentitions change during maturation. It is known, however, that some derived tyrannosaurids lack denticles as juveniles but gain them as adults (e.g., [128]), and so based on the ontogenetic change in absence/presence of denticles in these animals, the assumption that small theropods do not change their denticle morphologies (size and shape) during growth may be incorrect.

## Conclusion

### Theropod Tooth Lineages during the Late Cretaceous of North America

Larson and Currie [17] summarized the stratigraphic range of small theropod tooth morphotypes based on isolated teeth from the Santonian through the Maastrichtian, based on samples from the northern Rocky Mountain area. They indicated the presence of 23 quantitative morphotypes, up to eight of which were present at one time. They generally grouped these morphotypes into categories, with may or may not represent evolutionary lineages. Among these categories are (1) a “Saurornitholestinae” group that includes the late Campanian *Saurornitholestes langstoni* and the early Maastrichtian *Atrociraptor marshalli* (2) a Dromaeosauridae group that includes the late Campanian *Bambiraptor feinbergi*, (3) a “Dromaeosaurinae” group that includes the late Campanian *Dromaeosaurus albertensis*, (4) a “Dromaeosaurinae” group that includes the late Campanian *Zapsalis abradens*, (5) a troodontid group that includes the late Campanian *Troodon formosus*, (6) a troodontid group that includes *Pectinodon bakkeri*, (7) a group that includes the late Campanian *Richardoestesia gilmorei*, and (8) a group that includes the late Campanian *Richardoestesia isosceles*.

The recent description of new small theropod taxa that are not based exclusively on isolated teeth (e.g., *Acheroraptor*, *Talos*) do not necessarily contradict this hypothesis, and the presence of additional tooth morphotypes not mentioned or explicitly studied by Larson and Currie [17], such as *Paronychodon*, suggest at least a somewhat more complex and possibly diverse picture of small-toothed theropod evolutionary history in the Late Cretaceous of western North America, and perhaps indicate limitations of using primarily isolated theropod teeth to extrapolate large patterns.

Troodontids in particular may have been more diverse during the latest Cretaceous of North American than indicated by tooth taxa. Zanno et al. [143] named and described a small troodontid, *Talos sampsoni*, from the upper Campanian Kaiparowits Formation of southern Utah. *Talos* can be compared to other troodontid taxa represented by postcrania. However, it was not associated with craniodental remains that can be directly compared to the type specimen of *Troodon formosus*, an isolated tooth, nor can it be certain that isolated teeth from the Kaiparowits or other deposits are referable to this taxon [140], [143]. Larson and Currie [17] recognized a single *Troodon*-like troodontid lineage based on teeth, but the discovery of *Talos* is a reminder that skeletal remains (which are more diagnostic than most teeth) often reveal the presence of multiple taxa with a single lineage or small clade. Whether *Talos* has *Troodon*-like teeth is unknown at this point, but it would not be unexpected if it did. Regardless of whether that is the case, it is important to remember that a tooth lineage is not necessarily a single taxon. A lineage could represent multiple taxa over an interval of time (including ancestor-descendant pairs in an anagenetic sequence or sister taxa in a phylogenetic sequence).

Sankey [10] reported the presence of “*Troodon* sp. Large Morphology” from the late Maastrichtian of Montana (the specimen upon which this is based, UCMP 187178 is reported to be Paleocene based upon a specimen search of the UCMP database and we conclude that it likely reworked from the underlying Hell Creek Formation) that appears to be distinct from the troodontid *Pectinodon* that has been reported from the late Maastrichtian Lance and Hell Creek Formations of the northern Rocky Mountain region [20], [23]. Therefore it is probable that there is more than one lineage of troodontid in the latest Cretaceous of the northern Rocky Mountain region, and that this small-bodied theropod group may have been more diverse immediately before the end-Cretaceous extinction than previous suspected.

Dromaeosaurids may have also been more diverse in the latest Cretaceous of North America than indicated by the dental record, although recent evidence is equivocal on this point. Longrich [20] described a “Lance Dromaeosaurid” with “fang-like” teeth that lacked typical *Dromaeosaurus* characters such as a lingually twisted mesial carina and large mesial denticles (subequal in size to the denticles on the distal carina), or the distinctive apically-hooked distal denticles of *Saurornitholestes*, and possesses distinctive apicobasal ridges on the lingual and labial faces of the tooth crown. Evans et al. [20], [24] considered it likely that this tooth morphotype represent the isolated teeth of *Acheroraptor*, a taxon from the Upper Maastrichtian Hell Creek Formation that is represented by portions of the skull. Indeed, they concluded that most of the isolated dromaeosaurid teeth from the Hell Creek and Lance formations are likely attributable to *Acheroraptor*. However, some teeth from the large samples from the Hell Creek and Lance formations lack the distinctive apicobasal ridges, and it is therefore uncertain whether a lack of ridges indicates taxonomic or individual variation [24].

### San Juan Basin Record of Small Theropods

#### The Santonian

The sample of small theropod teeth from the Santonian Hosta Tongue of the Point Lookout Sandstone is small, but it comes from a very poorly sampled time interval in North America and appears to show the presence of a distinct small tyrannosauroid similar in tooth size and morphology to that from the similarly-aged Milk River Formation of Alberta [23], as well as a small dromaeosaurid that may be different from any previously reported from western North America. The Hosta Tongue and Milk River record the presence of one or more tyrannosauroids that existed near the middle of the “tyrannosaurid diversification interval” of the middle Late Cretaceous hypothesized by Loewen et al. [123]. The small size of the teeth from both these samples suggest the presence of tyrannosauroids smaller than the derived tyrannosaurids found in younger Late Cretaceous assemblages of the Western Interior [43], [123], although it is possible that all of the Hosta Tongue teeth come from very small juveniles.

#### The late Campanian

The San Juan Basin late Campanian record is much more extensive, and the four or more morphotypes we recognize closely resemble morphotypes reported from the late Campanian Dinosaur Park Formation of Alberta. These closely correspond to the *Saurornitholestes langstoni*, *Dromaeosaurus albertensis*, *Richardoestesia* spp. (but we are reluctant to recognize two distinct species of *Richardoestesia*), and *Troodon formosus* morphotypes. *Paronychodon* is also present, but it is not clear that it represents a valid taxon.

Previous workers have argued that troodontids are rare, or in some cases lacking, from southern North American Late Cretaceous dinosaur communities [19], [141], [144], [145]. Troodontids are present in the Campanian of southern Utah [32], [140], [143], but were thought to be absent from the Campanian of northwestern New Mexico and west Texas. Original reports of troodontids in the Aguja Formation [25] were later shown to be based on teeth of pachycephalosaurs [18]. Based on the specimens reported here, it is clear that troodontids are present, but rare, in Campanian strata of the San Juan Basin.

No teeth have yet been identified from the San Juan Basin that resemble either the dromaeosaurine *Zapsalis abradens* or the troodontid “*Pectinodon.*” Furthermore, the relative abundance of taxa differs from that of the Dinosaur Park Formation, with the teeth of a *Dromaeosaurus*-like taxon and troodontids being very rare in the Campanian of the San Juan Basin unlike their more common occurrence in the Dinosaur Park Formation.

Differences between late Campanian faunas of western North America have long been recognized based on the distribution of dinosaur taxa (e.g., [6], [8], [9], [146], [147]). These studies suggest that there was strong provinciality along the eastern edge of the landmass Laramidia that occupied the western margin of the Western Interior Seaway. Some studies argued for a north-south zonation of distinct faunal provinces [6], [8], [146], [148], possibly due to dispersal barriers [146], [149] that resulted in rapid diversification events among some dinosaur clades within restricted basins. This study adds additional evidence for faunal differences between a southern late Campanian vertebrate fauna and that of the northern Rocky Mountain region. The San Juan Basin small theropod Campanian fauna is similar in diversity to those reported from west Texas [18] and Utah [150], but is markedly less diverse than that of the Dinosaur Park Formation [e.g., 17]. This is similar to the pattern found for other groups of dinosaurs [6]. However, with that being said, we have been unable to identify any theropod tooth morphotype that is endemic to the San Juan Basin, and therefore we find no support for the hypothesis that any small theropods underwent a separate radiation in either the San Juan Basin or in the southern portion of Laramidia. We also note that the relatively smaller sample sizes from the Campanian of the San Juan Basin compared to those of the northern Rockies may explain some, or potentially all, of the diversity differences between the two regions.

#### The Late Maastrichtian

The sample of small theropod teeth from the upper Maastrichtian Naashoibito Member is small, but reveals important information on latest Cretaceous faunal diversity and beta diversity in western North America. The Naashoibito fauna contains tooth morphotypes that are similar to those reported from other latest Cretaceous faunas of North America. These are closely comparable to tooth morphotypes described by Larson and Currie [17] and include a tyrannosauroid that likely represents an early ontogenetic state of *Tyrannosaurus rex*, a dromaeosaurid (Dromaeosauridae Morphotype A) that is similar to the well-known “Saurornitholestinae” morphotype, a cf. “*Richardoestesia*” that is similar to the common *Richardoestesia gilmorei* or *R. isosceles* morphotype, and a troodontid, most similar to the widely-known cf. *Troodon* morphotype. At a finer level, however, comparisons between the San Juan Basin specimens and those from the northern Rocky Mountains reveal some similarities and some differences. Studies of northern Rockies faunas indicate the presence of at least one dromaeosaurid, probably equivalent to *Acheroraptor* [20], [24], two troodontids [20], [23] as well as one or two species of *Richardoestesia* [10], [17], [20].

Regarding dromaeosaurids, Evans et al. [24] argued that *Acheroraptor* represents the youngest dromaeosaurid and the only one present in the Lance or Hell Creek Formation of the northern Rocky Mountain region. The single dromaeosaurid tooth from the Naashoibito Member is similar to the “Saurornitholestine” morphotype described by Larson and Currie [17], but it lacks the apicobasal ridges on the labial face of the tooth observed in the holotype of *Acheroraptor* and the “Lance dromaeosaurid” [20]. It does bear low apicobasal ridges on the lingual face of the tooth, but unlike the ridged teeth of *Acheroratpor*, these are restricted to the basal half of the tooth crown. However, because apicobasal ridges may be variably present along the tooth row in *Acheroraptor*, we are uncertain if the Naashoibito taxon represents this or a distinct separate taxon. Quantitative tests are also difficult because the Naashoibito material is only a single tooth, but should become possible when sample sizes increase.

The Naashoibito troodontid is certainly distinct from *Pectinodon* from the Lance and Hell Creek formations [20] and probably different from the large troodontid described by Sankey [10] of the northern Rocky Mountain region. Thus at least three troodontid taxa were present at the end of the Cretaceous of western North America. The presence and high relative abundance of a troodontid in the Maastrichtian of the San Juan Basin is particularly interesting because troodontids are rare or absent in southern late Campanian vertebrate faunas of western North America (see above). It may also be different from a rare, large troodontid tooth morphotype known from Montana [10]. The Naashoibito troodontid is also distinct from the Campanian morphotype represented by the sample of “*Troodon formosus*” of the Dinosaur Park Formation and the early Maastrichtian “cf. *Troodon* sp.” from the Horseshoe Canyon Formation based on DFA and morphospace permutation tests. Its presence and high relative abundance relative to those of the late Campanian and early Maastrichtian and coeval faunas of the northern Rocky Mountain region is noteworthy. Its temporal and geographic separation, as well as morphological differences with other named western North American troodontids (e.g., *Troodon formosus*, *Pectinodon bakkeri*, and *Talos sampsoni*), make it likely that it represents a distinct taxon, one that is possibly endemic to the San Juan Basin, or at least the southern part of western North America.

The Naashoibito record also includes a small sample of a taxon that we tentatively refer to *Richardoestesia* sp. It is distinct from late Campanian cf. *Richardoestesia* from the San Juan Basin and more similar to those reported from other locales of the Western Interior in possessing mesial and distal denticles that are subequal in size.

Several workers have suggested that late Maastrichtian faunas of western North America, like those of the late Campanian, had marked faunal provinciality [7], [8], [9], with the San Juan Basin falling into a distinct *Alamosaurus* zone, characterized by the presence of the large titanosaurid sauropod *Alamosaurus* (compared to the rarity or absence of sauropods from more northern regions during this time). However, recent studies using multivariate statistical analyses based on records in the Paleobiology Database (PaleoDB.org) found no evidence to support distinct faunal regions of dinosaurs during the Maastrichtian of western North America [11]. With that said, we note here that the dataset Vavrek and Larsson [11] used appears to have some major flaws. For example, *Parasaurolophus*, *Daspletosaurus*, *Ankylosaurus*, *Monoclonius*, *Saurolophus*, *Pentaceratops*, and *Sphaerotholus* (Vavrek and Larsson, [11]; supplement sd01) are not known to be present in the Maastrichtian of New Mexico, despite what some records in the Paleobiology Database may indicate. Most of these are characteristic dinosaurs from the northern Rockies, and if their mistaken records in the southern faunas of New Mexico are indicative of a wider issue with the Paleobiology Database, it may be that an artificial signal of widely distributed dinosaur faunas emerges from multivariate analysis due to erroneous identifications of northern taxa in southern faunas.

The retreat of the Western Interior Seaway during the Maastrichtian may have allowed taxa to widen their geographic ranges, resulting in decreased endemism during this time compared to the late Campanian [149]. However, presence of a distinct and abundant troodontid in the Naashoibito Formation of the San Juan Basin, a taxon not present in northern latest Cretaceous faunas, at the very least indicate some differences between the small theropod faunas of northern and southern regions of North America during this time. Although sample sizes are small, the Naashoibito troodontid could provide some support for continued provinciality within western North America in the late Maastrichtian, at the time when the Chicxulub bolide hit and the most voluminous phase of Deccan volcanism occurred, right before the non-avian dinosaurs went extinct. It is interesting to note that Williamson and Weil [87] found similar support for provinciality in the Maastrichtian based on the relatively high abundance of the mammals *Glasbius* and *Essonodon* in the Naashoibito. These taxa are present, but rare in latest Cretaceous faunas of the northern Rocky Mountain region.

The small theropod fauna of the San Juan Basin may not provide any sweeping insights into the non-avian dinosaur extinction, but it does add new data to better understand how dinosaurs were distributed, and how they were changing, in North America during the few million years before the end of the Cretaceous. What is most striking is that there does not appear to be any major losses in small-bodied lineages across the Campanian-Maastrichtian. The Naashoibito record from the San Juan Basin includes the same suite of taxa that is common in the Campanian (and earlier): tyrannosauroids, dromaeosaurids, troodontids, and *Richardoestesia* (or a *Richardoestesia*-like taxon). No major components of the Campanian fauna are absent from the Maastrichtian assemblage, arguing against any major loss of theropod diversity during this time. A similar conclusion was recently presented by Gates et al. [136] based on small theropod teeth from the Hell Creek Formation, and this generally is consistent with regional and global patterns showing no clear declines in theropod diversity [5] or morphological disparity [1] over the final few million years of the Cretaceous. It may be that individual theropod lineages were becoming less diverse during the Maastrichtian (e.g., [24]), but teeth provide a clear record that the major components (clades/lineages) of small-bodied theropod diversity persisted deep into the Maastrichtian, most likely up to the Cretaceous-Paleogene boundary (see also [151]). Perhaps most importantly, the New Mexico record shows that the pattern observed in the Hell Creek is also true several thousand kilometers to the south, meaning that the well-sampled Hell Creek record may at least be representative of western North America as a whole when it comes to studying gross diversity patterns during the final days of the dinosaurs.

## Supporting Information

Appendix S1 **Measurements of small theropod teeth from the San Juan Basin, northwestern New Mexico.** FABL, fore-aft basal length; BW, basal width; CH, crown height; ADM, anterior denticles per millimeter; PDM, posterior denticles per millimeter. Measurements are in millimeters.
(XLSX)

Appendix S2 **Principal Component (PCA) and Discriminate Function Analyses (DFA) of small theropod teeth.**
(XLSX)
